# Supplementary material for: Using an integrative taxonomic approach to delimit a sibling species, Mycetomoellerius mikromelanos sp. nov. (Formicidae: Attini: Attina)
Source: PeerJ. 2021 Jun 24;9:e11622. doi: 10.7717/peerj.11622 (PMC8236233; doi:10.7717/peerj.11622)
Supplement: Supplemental Information 1 — In some studies, it was assumed that the smaller black species was M. zeteki sensu stricto (e.g., Adams et al., 2012a; Adams et al., 2012b) whereas in others uncertainty was indicated by referring to the smaller, darker species as M. cf. zeteki. Vouchers that are not represented by a physical ant specimens are indicated by (*) for GenBank depositions, (†) for fungal-cultivar GenBank deposition, and (‡) for other symbiont GenBank depositions [file peerj-09-11622-s001.docx]

**Appendix Table S1** –Published research on *Mycetomoellerius zeteki* and *M.*cf.*zeteki.* In some studies, it was assumed that the smaller black species was *M. zeteki* *sensu stricto* (e.g., Adams et al., 2012a, 2012b) whereas in others uncertainty was indicated by referring to the smaller, darker species as *M.*cf.*zeteki*. Vouchers that are not represented by a physical ant specimens are indicated by (*) for GenBank depositions, (†) for fungal-cultivar GenBank deposition, and (‡) for other symbiont GenBank depositions.

| **Published Name** | **Corrected Name** | **Material Included in this Study** | **Voucher Location** | **(Citation)**  **Journal** | **Corresponding Author** |
| --- | --- | --- | --- | --- | --- |
| ***T. zeteki & T. cf. zeteki*  2** | *M*. *mikromelanos* sp. nov. & *M. zeteki* | Yes | United States National Museum of Natural History | (Adams et al., 2012) Biochem Syst Ecol | Rachelle M.M. Adams |
| ***T. zeteki & T. cf. zeteki*** | *M*. *mikromelanos* sp. nov. & *M. zeteki* | Yes | United States National Museum of Natural History | (Adams et al., 2012) Eco Ento | Rachelle M.M. Adams |
| ***T. zeteki & T. cf. zeteki*** | *M*. *mikromelanos* sp. nov. & *M. zeteki* | Yes | United States National Museum of Natural History; Smithsonian Tropical Research Institute, Panama | (Adams et al., 2013) PNAS | Rachelle M.M. Adams and David R. Nash |
| ***T. zeteki*** | - | No | - | (Andersen et al., 2013) Mol Ecol | Sandra Breum Andersen and Jacobus J. Boomsma |
| ***T. cf. zeteki*** | - | No | Coleccion Nacional de Referencia Museo de Invertebrados, Universidad de Panama | (Armitage et al., 2012) Evolution | Sophie A. O. Armitage |
| ***T. cf. zeteki*** | - | No | - | (Baer & Boomsma, 2004) Behav Ecol | Boris Baer |
| ***T. cf. zeteki*** | - | No | - | (Baer et al., 2009) Behav Ecol | Boris Baer |
| ***T. zeteki*** | - | No | - | (Birnbaum & Gerardo, 2016) Am Nat | Nicole M. Gerardo |
| ***T. zeteki*** | - | No | - | (Boya et al., 2017) Sci Reports | Marcelino Gutérrez |
| ***T. zeteki*** | - | No | Museu de Zoologia da Universidadede São Paulo | (Brandão & Mayhé-Nunes, 2007) Mem Am Entomol Inst | C. Roberto F. Brandão and Antonio J. Mayhé-Nunes |
| ***T. zeteki*** | - | No | ‡ | (Cafaro & Currie, 2005) Can J Microbiol | Matias J. Cafaro |
| ***T. zeteki & T. cf. zeteki*** | - | No | * | (Cafaro et al., 2011) Proc R Soc B | Cameron R. Currie |
| ***T. cf. zeteki*** | - | No | - | (Currie et al., 1999) PNAS | Cameron R. Currie |
| ***T. cf. zeteki*** | - | No | - | (Cameron et al., 2003) Science | Cameron R. Currie |
| ***T. zeteki*** | - | No | United States National Museum of Natural History | (Cameron et al., 2006) Science | Cameron R. Currie |
| ***T. zeteki*** | - | No | * | (De Fine Licht & Boomsma, 2014) BMC Evo Bio | Henrik H. De Fine Licht |
| ***T. zeteki*** | - | No | - | (De Fine Licht et al., 2014) Nat Commun | Henrik H. De Fine Licht |
| ***T. cf. zeteki*** | *M. mikromelanos* sp. nov. | No | Natural History Museum of Denmark, Copenhagen | (Den Boer et al., 2010) Science | Jacobus Boomsma |
| ***T. cf. zeteki*** | - | No | Zoological Museum of the University of Copenhagen | (Dijkstra & Boomsma, 2008) Oikos | Michiel Dijkstra and Jacobus Boomsma |
| ***T. zeteki*** | *M. mikromelanos* sp. nov. | No | - | (Donoso, 2014) Ecography | David Donoso |
| ***T. zeteki*** | - | No | Coleccion Nacional de Referencia Museo de Invertebrados, Universidad de Panama | (Fernández-Marín et al., 2015) Proc R Soc B | Hermogenes Fernandez-Marin and Jacobus Boomsma |
| ***T. zeteki*** | - | No | Coleccion Nacional de Referencia Museo de Invertebrados, Universidad de Panama | (Fernández-Marín et al., 2013) Am Nat | Hermogenes Fernández-Marín and William Wcislo |
| ***T. zeteki*** | - | No | Zoological Museum, University of Puerto Rico; Smithsonian Tropical Research Institute, Panama | (Fernández-Marín et al., 2004) Biol J Linn Soc Lond | Hermogenes Fernández-Marín and William Wcislo |
| ***T. cf. zeteki*** | - | No | United States National Museum of Natural History; * | (Fernández-Marín et al., 2006) Proc R Soc B | William Wcislo |
| ***T. zeteki*** | - | No | Smithsonian Tropical Research Institute, Panama | (Fernández-Marín et al., 2009) Proc R Soc B | Hermogenes Fernández-Marín and William Wcislo |
| ***T. zeteki*** | - | No | - | (Fjerdingstad & Crozier, 2006) Am Nat | Else J. Fjerdingstad and Ross H. Crozier |
| ***T. zeteki & T. cf. zeteki*** | - | No | - | (Frost et al., 2010) Mol Ecol | Crystal Frost |
| ***T. cf. zeteki*** | - | No | - | (Hughes et al., 2008) Evolution | William O.H. Hughes |
| ***T. zeteki*** | - | ! | United States National Museum of Natural History | (Ješovnik et al., 2016) PloS ONE | Ana Jesovnik and Ted R. Schultz |
| ***T. zeteki*** | - |  | United States National Museum of Natural History; * | (Ješovnik et al., 2017) Syst Ent | Ana Jesovnik and Ted R. Schultz |
| ***T. zeteki*** | - | No | - | (Kaspari et al., 2012) Ecosphere | Mike Kaspari |
| ***T. zeteki & T. cf. zeteki*** | - | No | † | (Kooij et al., 2015) J Evo Bio | Pepijn Kooij & Dr. Jacobus Boomsma |
| ***T. zeteki & T. cf. zeteki*** | - | ! | ‡ | (Liberti et al., 2015) Mol Ecol | Rachelle M.M. Adams and Jacobus Boomsma |
| ***T. cf. zeteki*** | - | No | - | (Little & Currie, 2009) BMC Evo Bio | Cameron R. Currie |
| ***T. cf. zeteki*** | - | No | - | (Little et al., 2003) Naturissenschaften | Ainslie E. F. Little |
| ***T. cf. zeteki*** | - | No | - | (ALittle et al., 2006) Biol Lett | Ainslie E. F. Little |
| ***T. zeteki*** | - | No | Instituto Nacional de Biodiversidad, Costa Rica; Longino researach collection | (Longino & Colwell, 2011) Ecosphere | Jack Longino |
| ***T. zeteki*** | - | No | United States National Museum of Natural History | (Mangone & Currie, 2007) Can Entomol | Cameron R. Currie |
| ***T. zeteki*** | - | No | * | (Mueller et al., 2008) Evolution | Ulrich Mueller, Debadutta Dash, Christian Rabeling, and Andre Rodrigues |
| ***T. zeteki*** | - | Yes | * , National Museum of Natural History; University of Copenhagen | (Nygaard et al., 2016) Nature Comm | Sanne Nygaard, Guojie Zhang, and Jacobus Boomsma |
| ***T. cf. zeteki*** | - | No | Coleccion Nacional de Referencia Museo de Invertebrados, Universidad de Panama | (Pérez-Ortega et al., 2010) Ins Soc | Hermogenes Fernandez-Marin and William Wcislo |
| ***T. zeteki & T. cf. zeteki*** | - | No | - | (Poulsen & Currie, 2010) PloS ONE | Michael Poulsen |
| ***T. zeteki*** | - | No | ‡ | (Poulsen et al., 2007) PloS ONE | Michael Poulsen |
| ***T. zeteki*** | - | No | - | (Sapountzis et al., 2018) eLife | Panagiotis Sapountzis and Jacobus J Boomsma |
| ***T. cf. zeteki*** | - | No | United States National Museum of Natural History | (Schultz & Brady, 2008) PNAS | Ted Schultz |
| ***T. zeteki*** | - | No | † | (Scott et al., 2009) Mol Ecol Resour | Ulrich Mueller |
| ***T. zeteki & T. cf. zeteki 3*** | - | No | - | (Seal, 2009) Insect Soc | John Seal |
| ***T. zeteki*** | - | No | Coleccion Nacional de Referencia Museo de Invertebrados, Universidad de Panama; Smithsonian Tropical Research Institute, Panama | (Seid et al., 2011) Brain Behav Evo | Wiliam Wcisclo |
| ***T. cf. zeteki*** | - | No | † | (Semenova et al., 2011) BMC Microbiology | Morten Schiøtt |
| ***T. zeteki*** | - | No | ‡ | (Sen et al., 2009) PNAS | Ruchira Sen and Ulrich Mueller |
| ***T. zeteki*** | - | No | - | (Shik et al., 2014) Am Nat | Johnathan Shik |
| ***T. zeteki*** | Likely *M. atlanticus* | No | Museu de Zoologia da Universidadede São Paulo | (Silva et al., 2007) Forest Eco Manage | Rogério Silva |
| ***M. sp. nov. & M. zeteki*** | *M*. *mikromelanos* sp. nov. & *M. zeteki* | Yes | Museu de Zoologia da Universidade de São Paulo; National Museum of Natural History | (Solomon et al., 2019) Syst Ent | Scott E Solomon, Christian Rabeling, Ted Schultz |
| ***T. zeteki*** | - | No | ‡ | (Taerum et al., 2007) Proc R Soc B | Cameron R. Currie |
| ***T. cf. zeteki*** | - | No | Museo de Entomología de la Universidad del Valle | (Valdés-Rodríguez et al., 2014) Rev Biol Trop | Sephany Valdes-Rodriguez, Patricia Chacon de Ulloa, and Inge Armbrecht |
| ***T. zeteki*** | - | No | Museo Entomológico Universidad Nacional Agronomía Bogotá | (Vergara-Navarro & Serna, 2013) Agron Colombian | Francisco Serna |
| ***T. cf. zeteki*** | - | No | United States National Museum of Natural History | (Villesen et al., 2002) Proc R Soc B | Jacobus Boomsma |
| ***T. cf. zeteki*** | - | No | United States National Museum of Natural History | (Villesen & Boomsma, 2003) Behav Ecol Sociobiol | Jacobus Boomsma |
| ***T. cf. zeteki*** | - | No | - | (Villesen et al., 2002) Mol Ecol Notes | Jacobus Boomsma |
| ***T. cf. zeteki*** | - | No | ‡ | (Wallace et al., 2014) Microbiol | Adrián Pinto Tomás |
| ***T. zeteki*** | *M. zeteki* | Yes | United States National Museum of Natural History | (Weber, 1940) Revista de Ento | N/A |
| ***T. zeteki*** | - | No | United States National Museum of Natural History; * | (Wetterer et al., 1998) Mol Phylo Evo | James K Wetterer |
| ***T. zeteki*** | - | No | - | (Zhang et al., 2007) ISME J | Cameron R. Currie |

**References**

Adams, R. M. M., Jones, T. H., Jeter, A. W., De Fine Licht, H. H., Schultz, T. R., & Nash, D. R. (2012). A comparative study of exocrine gland chemistry in *Trachymyrmex* and *Sericomyrmex* fungus-growing ants. *Biochemical Systematics and Ecology*, *40*(February 2012), 91–97. https://doi.org/10.1016/j.bse.2011.10.011

Adams, R. M. M., Liberti, J., Illum, A. A., Jones, T. H., Nash, D. R., & Boomsma, J. J. (2013). Chemically armed mercenary ants protect fungus-farming societies. *Proceedings of the National Academy of Sciences*, *110*(39), 15752–15757. https://doi.org/10.1073/pnas.1311654110

Adams, R. M. M., Shah, K., Antonov, L. D., & Mueller, U. G. (2012). Fitness consequences of nest infiltration by the mutualist-exploiter *Megalomyrmex adamsae*. *Ecological Entomology*, *37*(6), 453–462. https://doi.org/10.1111/j.1365-2311.2012.01384.x

Andersen, S. B., Hansen, L. H., Sapountzis, P., Sørensen, S. J., & Boomsma, J. J. (2013). Specificity and stability of the *Acromyrmex*-*Pseudonocardia* symbiosis. *Molecular Ecology*, *22*(16), 4307–4321. https://doi.org/10.1111/mec.12380

Armitage, S. A. O., Wcislo, W. T., & Boomsma, J. J. (2012). An evaluation of the possible adaptive function of fungal brood covering by Attine ants. *Evolution*, *66*(6), 1966–1975. https://doi.org/10.5061/dryad.r36d6k6t

Baer, B., & Boomsma, J. J. (2004). Male reproductive investment and queen mating-frequency in fungus-growing ants. *Behavioral Ecology*, *15*(3), 426–432. https://doi.org/10.1093/beheco/arh025

Baer, B., Dijkstra, M. B., Mueller, U. G., Nash, D. R., & Boomsma, J. J. (2009). Sperm length evolution in the fungus-growing ants. *Behavioral Ecology*, *20*(1), 38–45. https://doi.org/10.1093/beheco/arn112

Birnbaum, S. S. L., & Gerardo, N. M. (2016). Patterns of specificity of the pathogen *Escovopsis* across the fungus-growing ant symbiosis. *American Naturalist*, *188*(1), 52–65. https://doi.org/10.1086/686911

Boya, C. A., Fernández-Marín, H., Mejiá, L. C., Spadafora, C., Dorrestein, P. C., & Gutiérrez, M. (2017). Imaging mass spectrometry and MS/MS molecular networking reveals chemical interactions among cuticular bacteria and pathogenic fungi associated with fungus-growing ants. *Scientific Reports*, *7*(1), 1–13. https://doi.org/10.1038/s41598-017-05515-6

Brandão, C. R. F., & Mayhé-Nunes, A. J. (2007). A phylogenetic hypothesis for the *Trachymyrmex* species groups, and the transition from fungus-growing to leaf-cutting in the Attini. *Memoirs of the American Entomological Institut*, *80*, 73–87. https://doi.org/10.1533/9781845696382.2.267

Cafaro, M. J., & Currie, C. R. (2005). Phylogenetic analysis of mutualistic filamentous bacteria associated with fungus-growing ants. *Canadian Journal of Microbiology*, *51*(6), 441–446. https://doi.org/10.1139/w05-023

Cafaro, M. J., Poulsen, M., Little, A. E. F., Price, S. L., Gerardo, N. M., Wong, B., Stuart, A. E., Larget, B., Abbot, P., & Currie, C. R. (2011). Specificity in the symbiotic association between fungus-growing ants and protective *Pseudonocardia* bacteria. *Proceedings of the Royal Society B: Biological Sciences*, *278*(1713), 1814–1822. https://doi.org/10.1098/rspb.2010.2118

Currie, C. R., Mueller, U. G., & Malloch, D. (1999). The agricultural pathology of ant fungus gardens. *Proceedings of the National Academy of Sciences of the United States of America*, *96*(14), 7998–8002. http://www.pubmedcentral.nih.gov/articlerender.fcgi?artid=22176&tool=pmcentrez&rendertype=abstract

Currie, Cameron R., Wong, B., Stuart, A. E., Schultz, T. R., Rehner, S. A., Mueller, U. G., Sung, G. H., Spatafora, J. W., & Straus, N. A. (2003). Ancient tripartite coevolution in the attine ant-microbe symbiosis. *Science*, *299*(5605), 386–388. https://doi.org/10.1126/science.1078155

Currie, Cameron R, Poulsen, M., Mendenhall, J., Boomsma, J. J., & Billen, J. (2006). Coevolved crypts and exocrine glands support mutualistic bacteria in fungus-growing ants. *Science*, *311*(28), 81–83. https://doi.org/10.1126/science.1119744

De Fine Licht, H. H., & Boomsma, J. J. (2014). Variable interaction specificity and symbiont performance in Panamanian *Trachymyrmex* and *Sericomyrmex* fungus-growing ants. *BMC Evolutionary Biology*, *14*(1), 244. https://doi.org/10.1186/s12862-014-0244-6

De Fine Licht, H. H., Boomsma, J. J., & Tunlid, A. (2014). Symbiotic adaptations in the fungal cultivar of leaf-cutting ants. *Nature Communications*, *5*(5675), 1–10. https://doi.org/10.1038/ncomms6675

Den Boer, S. P. A., Baer, B., Boomsma, J. J., Smith, J. M., & Biol, T. (2010). Seminal fluid mediates ejaculate competition in social insects. *Science*, *327*(5972), 1506–1509. https://doi.org/10.1126/science.1184709

Dijkstra, M. B., & Boomsma, J. J. (2008). Sex allocation in fungus-growing ants: Worker or queen control without symbiont-induced female bias. *Oikos*, *117*(12), 1892–1906. https://doi.org/10.1111/j.1600-0706.2008.16822.x

Donoso, D. A. (2014). Assembly mechanisms shaping tropical litter ant communities. *Ecography*, *37*(5), 490–499. https://doi.org/10.1111/j.1600-0587.2013.00253.x

Elizondo Wallace, D. E., Vargas Asensio, J. G., & Pinto Tomás, A. A. (2014). Correlation between virulence and genetic structure of *Escovopsis* strains from leaf-cutting ant colonies in Costa Rica. *Microbiology*, *160*, 1727–1736. https://doi.org/10.1099/mic.0.073593-0

Fernández-Marín, H., Zimmerman, J. K., & Wcislo, W. T. (2004). Ecological traits and evolutionary sequence of nest establishment in fungus-growing ants (Hymenoptera, Formicidae, Attini). *Biological Journal of the Linnean Society*, *81*(1), 39–48. https://doi.org/10.1111/j.1095-8312.2004.00268.x

Fernández-Marín, H, Zimmerman, J. K., Rehner, S. A., & Wcislo, W. T. (2006). Active use of the metapleural glands by ants in controlling fungal infection. *Proceedings of the Royal Society B Biology*, *273*(1594), 1689–1695.

Fernández-Marín, Hermógenes, Bruner, G., Gomez, E. B., Nash, D. R., Boomsma, J. J., & Wcislo, W. T. (2013). Dynamic disease management in *Trachymyrmex* fungus-growing ants (Attini: Formicidae). *The American Naturalist*, *181*(4), 571–582. https://doi.org/10.1086/669664

Fernández-Marín, Hermógenes, Nash, D. R., Higginbotham, S., Estrada, C., Van Zweden, J. S., D’Ettorre, P., Wcislo, W. T., & Boomsma, J. J. (2015). Functional role of phenylacetic acid from metapleural gland secretions in controlling fungal pathogens in evolutionarily derived leaf-cutting ants. *Proceedings of the Royal Society B: Biological Sciences*, *282*(1807), 1–9. https://doi.org/10.1098/rspb.2015.0212

Fernández-Marín, Hermógenes, Zimmerman, J. K., Nash, D. R., Boomsma, J. J., & Wcislo, W. T. (2009). Reduced biological control and enhanced chemical pest management in the evolution of fungus farming in ants. *Proceedings of the Royal Society B Biology*, *276*, 2263–2269. https://doi.org/10.1098/rspb.2009.0184

Fjerdingstad, E. J., & Crozier, R. H. (2006). The evolution of worker caste diversity in social insects. *American Naturalist*, *167*(3), 390–400. https://doi.org/10.1086/499545

Frost, C. L., Fernández-Marín, H., Smith, J. E., & Hughes, W. O. H. (2010). Multiple gains and losses of *Wolbachia* symbionts across a tribe of fungus-growing ants. *Molecular Ecology*, *19*(18), 4077–4085. https://doi.org/10.1111/j.1365-294X.2010.04764.x

Hughes, W. O. H., Pagliarini, R., Madsen, H. B., Dijkstra, M. B., & Boomsma, J. J. (2008). Antimicrobial defense shows an abrupt evolutionary transition in the fungus-growing ants. *Evolution; International Journal of Organic Evolution*, *62*(5), 1252–1257. https://doi.org/10.1111/j.1558-5646.2008.00347.x

Ješovnik, A., González, V. L., & Schultz, T. R. (2016). Phylogenomics and divergence dating of fungus-farming ants (Hymenoptera: Formicidae) of the genera *Sericomyrmex* and *Apterostigma*. *PLoS ONE*, *11*(7), 1–18. https://doi.org/10.1371/journal.pone.0151059

Ješovnik, A., Sosa-Calvo, J., Lloyd, M. W., Branstetter, M. G., Fernández, F., & Schultz, T. R. (2017). Phylogenomic species delimitation and host-symbiont coevolution in the fungus-farming ant genus *Sericomyrmex* Mayr (Hymenoptera: Formicidae): ultraconserved elements (UCEs) resolve a recent radiation. *Systematic Entomology*, *42*(3), 523–542. https://doi.org/10.1111/syen.12228

Kaspari, M., Donoso, D., Lucas, J. A., Zumbusch, T., & Kay, A. D. (2012). Using nutritional ecology to predict community structure: a field test in Neotropical ants. *Ecosphere*, *3*(11), 1–15. http://www.esajournals.org/doi/abs/10.1890/ES12-00136.1

Kooij, P. W., Aanen, D. K., Schiøtt, M., & Boomsma, J. J. (2015). Evolutionarily advanced ant farmers rear polyploid fungal crops. *Journal of Evolutionary Biology*, *28*(11), 1911–1924. https://doi.org/10.1111/jeb.12718

Liberti, J., Sapountzis, P., Hansen, L. H., Sørensen, S. J., Adams, R. M. M., & Boomsma, J. J. (2015). Bacterial symbiont sharing in Megalomyrmex social parasites and their fungus-growing ant hosts. *Molecular Ecology*, *24*, 3151–3169. https://doi.org/10.1111/mec.13216

Little, A. E., & Currie, C. R. (2009). Parasites may help stabilize cooperative relationships. *BMC Evolutionary Biology*, *9*(1), 1–9. https://doi.org/10.1186/1471-2148-9-124

Little, A. E. F., Murakami, T., Mueller, U. G., & Currie, C. R. (2003). The infrabuccal pellet piles of fungus-growing ants. *Die Naturwissenschaften*, *90*(12), 558–562. https://doi.org/10.1007/s00114-003-0480-x

Little, A. E. F., Murakami, T., Mueller, U. G., & Currie, C. R. (2006). Defending against parasites: fungus-growing ants combine specialized behaviours and microbial symbionts to protect their fungus gardens. *Biology Letters*, *2*(2006), 12–16. https://doi.org/doi:10.1098/rsbl.2005.0371

Longino, J. T., & Colwell, R. K. (2011). Density compensation, species composition, and richness of ants on a neotropical elevational gradient. *Ecosphere*, *2*(3), 1–20. https://doi.org/10.1890/ES10-00200.1

Mangone, D. M., & Currie, C. R. (2007). Garden substrate preparation behaviours in fungus-growing ants. *The Canadian Entomologist*, *139*(6), 841–849. https://doi.org/http://esc-sec.org/canent1.htm

Mueller, U. G., Dash, D., Rabeling, C., & Rodrigues, A. (2008). Coevolution between attine ants and actinomycete bacteria: a reevaluation. *Evolution*, *62*(11), 2894–2912. https://doi.org/10.1111/j.1558-5646.2008.00501.x

Nygaard, S., Hu, H., Li, C., Schiøtt, M., Chen, Z., Yang, Z., Xie, Q., Ma, C., Deng, Y., Dikow, R., Rabeling, C., Nash, D. R., Wcislo, W. T., Brady, S. G., Schultz, T. R., Zhang, G., & Boomsma, J. J. (2016). Reciprocal genomic evolution in the ant-fungus agricultural symbiosis. *Nature Communications*, *7*(12233), 1–9. https://doi.org/10.1038/ncomms12233

Pérez-Ortega, B., Fernández-Marín, H., Loiácono, M. S., Galgani, P., & Wcislo, W. T. (2010). Biological notes on a fungus-growing ant, *Trachymyrmex* cf. *zeteki* (Hymenoptera, Formicidae, Attini) attacked by a diverse community of parasitoid wasps (Hymenoptera, Diapriidae). *Insectes Sociaux*, *57*(3), 317–322. https://doi.org/10.1007/s00040-010-0086-1

Poulsen, M., & Currie, C. R. (2010). Symbiont interactions in a tripartite mutualism: exploring the presence and impact of antagonism between two fungus-growing ant mutualists. *PloS One*, *5*(1), e8748. https://doi.org/10.1371/journal.pone.0008748

Poulsen, M., Erhardt, D. P., Molinaro, D. J., Lin, T.-L., & Currie, C. R. (2007). Antagonistic bacterial interactions help shape host-symbiont dynamics within the fungus-growing ant-microbe mutualism. *PLoS ONE*, *2*(9), 1–15. https://doi.org/10.1371/journal.pone.0000960

Sapountzis, P., Zhukova, M., Shik, J. Z., Schiott, M., & Boomsma, J. J. (2018). Reconstructing the functions of endosymbiotic mollicutes in fungus-growing ants. *ELife*, *7*, 1–31. https://doi.org/10.7554/eLife.39209

Schultz, T. R., & Brady, S. G. (2008). Major evolutionary transitions in ant agriculture. *Proceedings of the National Academy of Sciences*, *105*(14), 5435–5440. https://doi.org/10.1073/pnas.0711024105

Scott, J. J., Weskin, M. K., Cooper, M., & Mueller, U. G. (2009). Polymorphic microsatellite markers for the symbiotic fungi cultivated by leaf cutter ants (Attini, Formicidae). *Molecular Ecology Resources*, *9*, 1391–1394. https://doi.org/10.3182/20120912-3-BG-2031.00041

Seal, J. N. (2009). Scaling of body weight and fat content in fungus-gardening ant queens: does this explain why leaf-cutting ants found claustrally? *Insectes Sociaux*, *56*(2), 135–141. https://doi.org/10.1007/s00040-009-0002-8

Seid, M. A., Castillo, A., & Wcislo, W. T. (2011). The allometry of brain miniaturization in ants. *Brain, Behavior and Evolution*, *77*(1), 5–13. https://doi.org/10.1159/000322530

Semenova, T. A., Hughes, D. P., Boomsma, J. J., & Schiøtt, M. (2011). Evolutionary patterns of proteinase activity in attine ant fungus gardens. *BMC Microbiology*, *11*(15), 1–11. https://doi.org/10.1186/1471-2180-11-15

Sen, R., Ishak, H. D., Estrada, D., Dowd, S. E., Hong, E., & Mueller, U. G. (2009). Generalized antifungal activity and 454-screening of Pseudonocardia and Amycolatopsis bacteria in nests of fungus-growing ants. *Proceedings of the National Academy of Sciences*, *106*(42), 17805–17810. https://doi.org/10.1073/pnas.0904827106

Shik, J. Z., Santos, J. C., Seal, J. N., Kay, A., Mueller, U. G., & Kaspari, M. (2014). Metabolism and the rise of fungus cultivation by ants. *The American Naturalist*, *184*(3), 364–373. https://doi.org/10.1086/677296

Silva, R. R., Feitosa, R. S. M., & Eberhardt, F. (2007). Reduced ant diversity along a habitat regeneration gradient in the southern Brazilian Atlantic Forest. *Forest Ecology and Management*, *240*(1–3), 61–69. https://doi.org/10.1016/j.foreco.2006.12.002

Solomon, S. E., Rabeling, C., Sosa-Calvo, J., Lopes, C. T., Rodrigues, A., Vasconcelos, H. L., Bacci, M., Mueller, U. G., & Schultz, T. R. (2019). The molecular phylogenetics of *Trachymyrmex* Forel ants and their fungal cultivars provide insights into the origin and coevolutionary history of ‘higher-attine’ ant agriculture. *Systematic Entomology*, *44*(4), 939–956. https://doi.org/10.1111/syen.12370

Taerum, S. J., Cafaro, M. J., Little, A. E. F., Schultz, T. R., & Currie, C. R. (2007). Low host-pathogen specificity in the leaf-cutting ant-microbe symbiosis. *Proceedings of the Royal Society B: Biological Sciences*, *274*(1621), 1971–1978. https://doi.org/10.1098/rspb.2007.0431

Valdés-Rodríguez, S., Chacón de Ulloa, P., & Armbrecht, I. (2014). Especies de hormigas del suelo en el Parque Nacional Natural Gorgona, Pacífico Colombiano. *Revista de Biología Tropical*, *62*(February), 265. https://doi.org/10.15517/rbt.v62i0.16340

Vergara-Navarro, E., & Serna, F. (2013). A checklist of the ants (Hymenoptera: Formicidae) of the department of Antioquia, Colombia and new records for the country. *Agronomía Colombiana*, *31*(3), 324–342.

Villesen, P., & Boomsma, J. J. (2003). Patterns of male parentage in the fungus-growing ants. *Behavioral Ecology and Sociobiology*, *53*(4), 246–253. https://doi.org/10.1007/s00265-002-0577-9

Villesen, P, Gertsch, P. J., & Boomsma, J. J. (2002). Microsatellite primers for fungus growing ants. *Molecular Ecology Notes*, *2*, 320–322. https://doi.org/10.1046/j.l471-8278

Villesen, Palle, Murakami, T., Schultz, T. R., & Boomsma, J. J. (2002). Identifying the transition between single and multiple mating of queens in fungus-growing ants. *Proceedings of the Royal Society London B Biology*, *269*(1500), 1541–1548. https://doi.org/10.1098/rspb.2002.2044

Weber, N. A. (1940). The biology of the fungus-growing ants. Part VI. Key to *Cyphomyrmex*, new Attini and a new guest ant. *Revista de Entomologia, Rio de Janeiro:*, *11*, 406–427. https://doi.org/10.5281/zenodo.25008

Wetterer, J. K., Schultz, T. R., & Meier, R. (1998). Phylogeny of fungus-growing ants (Tribe Attini) based on mtDNA sequence and morphology. *Molecular Phylogenetics and Evolution*, *9*(1), 42–47. https://doi.org/10.1006/mpev.1997.0466

Zhang, M. M., Poulsen, M., & Currie, C. R. (2007). Symbiont recognition of mutualistic bacteria by *Acromyrmex* leaf-cutting ants. *ISME Journal*, *1*(4), 313–320. https://doi.org/10.1038/ismej.2007.41
