# Supplementary material for: Using an integrative taxonomic approach to delimit a sibling species, Mycetomoellerius mikromelanos sp. nov. (Formicidae: Attini: Attina)
Source: PeerJ. 2021 Jun 24;9:e11622. doi: 10.7717/peerj.11622 (PMC8236233; doi:10.7717/peerj.11622)
Supplement: Supplemental Information 2 — (*) Indicates new genera. [file peerj-09-11622-s002.docx]

| Genera | | New species in Genera | Reference |
| --- | --- | --- | --- |
| *Atta* | 1 | | (Fontenla, 1995) |
| *Acromyrmex* | 4 | | (Bolton, 1995; Schultz et al., 1998; De Souza et al., 2007; Rabeling et al., 2015) |
| *Apterostigma* | 24 | | (Lattke, 1997, 1999; Schultz, 2007) |
| *Cyatta* * | 1 | | (Sosa-Calvo et al., 2013) |
| *Cyphomyrmex* | 3 | | (Schultz et al., 2002; de Andrade, 2003) |
| *Kalathomyrmex ** | 1 | | (Klingenberg & Brandão, 2009) |
| *Mycetagroicus ** | 4 | | (Brandão & Mayhé-Nunes, 2001, 2008) |
| *Mycetarotes* | 2 | | (Mayhé-Nunes, 1995) |
| *Mycetomoellerius* * | 4 | | (Mayhé-Nunes & Brandão, 2002, 2005, 2007) |
| *Mycetophylax* | 2 | | (Mackay & Serna, 2010) |
| *Mycetosoritis* | 1 | | (Mackay, 1998) |
| *Mycocepurus* | 1 | | (Rabeling & Bacci, 2010) |
| *Myrmicocrypta* | 3 | | (Sosa-Calvo & Schultz, 2010) |
| *Sericomyrmex* | 3 | | (Ješovnik & Schultz, 2017) |
| *Trachymyrmex* | 3 | | (Mackay & Mackay, 1997; Rabeling et al., 2007; Sánchez-Peña et al., 2017) |
| *Xerolitor ** | 1 | | (Sosa-Calvo et al., 2018) |
| Total……………………..........58 | | | (antweb.org, 2019) |

**Table S1** – New fungus-growing ant species described since from 1995 until July 2019. (*) Indicates new genera.
