# Supplementary material for: Using an integrative taxonomic approach to delimit a sibling species, Mycetomoellerius mikromelanos sp. nov. (Formicidae: Attini: Attina)
Source: PeerJ. 2021 Jun 24;9:e11622. doi: 10.7717/peerj.11622 (PMC8236233; doi:10.7717/peerj.11622)
Supplement: Supplemental Information 3 [file peerj-09-11622-s003.docx]

| **Taxon** | **DNAex** | **Voucher** | **EF1a-F1** | **EF1a-F2** | **LwRh** | **wg** | **COI** |
| --- | --- | --- | --- | --- | --- | --- | --- |
| *M. mikromelanos sp. nov.* | 312 | UGM951118-02 | EU204339 | EU204580 | EU204505 | MK600239 | – |
| *M. mikromelanos sp. nov.* | 449 | RMMA050801-08 | MK600330 | MK600004 | MK600145 | MK600240 | MK600077 |
| *M. mikromelanos sp. nov.* | 450 | RMMA050727-06 | MK600331 | MK600005 | MK600146 | MK600241 | MK600078 |
| *M. mikromelanos sp. nov.* | 575 | JSC030826-01 | MK600332 | MK600006 | MK600147 | MK600242 | MK600079 |
| *M. mikromelanos sp. nov.* | 858 | RMMA090930-09 | MK600334 | MK600008 | MK600149 | MK600244 | MK600081 |
| *M. mikromelanos sp. nov.* | 862 | RMMA050105-29 | MK600335 | MK600009 | MK600150 | MK600245 | MK600082 |
| *M. zeteki* | 451 | RMMA050816-04 | MK600342 | MK600016 | MK600157 | MK600253 | MK600089 |
| *M. zeteki* | A444 | RMMA050818-05 | MK600343 | MK600017 | MK600158 | MK600254 | – |
| *M. turrifex* | 571 | CR071225-02 | MK600379 | MK600054 | MK600192 | MK600304 | MK600120 |
| *M. turrifex* | T25 | CR051012-01 | MK600380 | MK600055 | MK600193 | MK600305 | – |

**Table S2** - GenBank accession numbers for samples used in Solomon et al. (2019).
