# Supplementary material for: Using an integrative taxonomic approach to delimit a sibling species, Mycetomoellerius mikromelanos sp. nov. (Formicidae: Attini: Attina)
Source: PeerJ. 2021 Jun 24;9:e11622. doi: 10.7717/peerj.11622 (PMC8236233; doi:10.7717/peerj.11622)
Supplement: Supplemental Information 4 — Scaffolds found when the consensus sequences were blasted to the published M. zeteki genome. [file peerj-09-11622-s004.docx]

| **Gene** | **Scaffold** | **Query Coverage (blastn)** |
| --- | --- | --- |
| cytochrome oxidase 1 (COI) | NW_017256612.1 | >95% |
| cytochrome oxidase 1 (COI) | NW_017257461.1 | >95% |
| cytochrome oxidase 1 (COI) | NW_017257978.1 | >95% |
| cytochrome oxidase 1 (COI) | NW_017257810.1 | >95% |
| cytochrome oxidase 1 (COI) | NW_017257228.1 | >95% |
| cytochrome oxidase 1 (COI) | NW_017257054.1 | <95% |
| cytochrome oxidase 1 (COI) | NW_017257980.1 | <95% |
| cytochrome oxidase 1 (COI) | NW_017256483.1 | <95% |
| cytochrome oxidase 1 (COI) | NW_017256149.1 | <95% |
| cytochrome oxidase 1 (COI) | NW_017256991.1 | <95% |
| cytochrome oxidase 1 (COI) | NW_017257728.1 | <95% |
| cytochrome oxidase 1 (COI) | NW_017257588.1 | <95% |
| Elongation factor 1a (F1) | NW_017257294.1 | 100% |
| Elongation factor 1a F2 copy (F2) | NW_017257294.1 | 100% |
| Long-wavelength rhodopsin (LWRh) | NW_017256212.1 | 100% |
| Wingless (WG) | NW_017256287.1 | 100% |

**Table S3** - Scaffolds found when we blasted the consensus sequence to the published *M. zeteki* genome.
