# Supplementary material for: Using an integrative taxonomic approach to delimit a sibling species, Mycetomoellerius mikromelanos sp. nov. (Formicidae: Attini: Attina)
Source: PeerJ. 2021 Jun 24;9:e11622. doi: 10.7717/peerj.11622 (PMC8236233; doi:10.7717/peerj.11622)
Supplement: Supplemental Information 5 — Table of partitioned measurements for examined Mycetomoellerius mikromelanos sp. nov. (mikro.) and M. zeteki (zeteki) specimen by caste and species, including standard errors (SE), minimum and maximum standard measurements in millimeters, except for indices. The junior synonym of M. zeteki is included as “balboai” which are included in the entire M. zeteki partition. [file peerj-09-11622-s005.docx]

| *mikro.* worker  N=117  (28 nests) | HW | HL | SL | EL | FL | ML | PL | PPL | GL | CI | EI | SI | FLI | WaL | TL |
| --- | --- | --- | --- | --- | --- | --- | --- | --- | --- | --- | --- | --- | --- | --- | --- |
| Mean | 1.123 | 0.986 | 1.031 | 0.201 | 0.695 | 1.657 | 0.409 | 0.254 | 1.096 | 114.007 | 20.369 | 91.829 | 61.842 | 0.664 | 4.402 |
| SE | 0.004 | 0.004 | 0.004 | 0.001 | 0.003 | 0.007 | 0.004 | 0.002 | 0.005 | 0.339 | 0.111 | 0.226 | 0.174 | 0.005 | 0.017 |
| Min – Max Range | 1.012-1.215 | 0.825-1.121 | 0.904-1.197 | 0.165-0.230 | 0.620-0.810 | 1.441-1.911 | 0.310-0.507 | 0.225-0.293 | 0.947-1.197 | 102.761-123.517 | 17.690-22.820 | 87.792-100.194 | 57.547-68.853 | 0.551-0.762 | 3.843-4.877 |
| *zeteki* worker  N=54  (13 nests) | **HW** | **HL** | **SL** | **EL** | **FL** | **ML** | **PL** | **PPL** | **GL** | **CI** | **EI** | **SI** | **FLI** | **WaL** | **TL** |
| Mean | 1.435 | 1.175 | 1.166 | 0.256 | 0.862 | 1.972 | 0.482 | 0.298 | 1.255 | 122.141 | 21.831 | 81.329 | 60.046 | 0.780 | 5.181 |
| SE | 0.013 | 0.011 | 0.009 | 0.002 | 0.010 | 0.018 | 0.007 | 0.004 | 0.014 | 0.390 | 0.141 | 0.311 | 0.274 | 0.009 | 0.046 |
| Min – Max Range | 1.208-1.602 | 1.012-1.330 | 1.040-1.360 | 0.207-0.286 | 0.714-1.020 | 1.665-2.248 | 0.385-0.605 | 0.253-0.363 | 1.043-1.520 | 115.790-127.767 | 17.975-24.280 | 77.441-86.661 | 55.287-67.208 | 0.647-0.957 | 4.520-6.043 |
| *balboai* worker  N=3 | HW | HL | SL | EL | FL | ML | PL | PPL | GL | CI | EI | SI | FLI | WaL | TL |
| Mean | 1.520 | 1.216 | 1.227 | 0.259 | 0.904 | 2.089 | 0.456 | 0.308 | 1.292 | 125.012 | 21.258 | 80.720 | 59.515 | 0.764 | 5.36 |
| SE | 0.021 | 0.012 | 0.026 | 0.015 | 0.008 | 0.060 | 0.012 | 0.004 | 0.029 | 1.436 | 1.102 | 0.829 | 0.398 | 0.013 | 0.078 |
| Min – Max Range | 1.459-1.550 | 1.185-1.246 | 1.155-1.276 | 0.213-0.274 | 0.881-0.912 | 1.915-2.188 | 0.425-0.486 | 0.304-0.319 | 1.246-1.368 | 121.99-127.467 | 17.975-22.533 | 79.164-82.323 | 58.839-60.384 | 0.729-0.790 | 5.136-5.486 |
| *mikro.* gyne  N=25  (9 nests) | **HW** | **HL** | **SL** | **EL** | **FL** | **ML** | **PL** | **PPL** | **GL** | **CI** | **EI** | **SI** | **FLI** | **WaL** | **TL** |
| Mean | 1.298 | 1.142 | 1.036 | 0.270 | 0.833 | 2.028 | 0.599 | 0.312 | 1.579 | 113.716 | 23.619 | 79.847 | 64.166 | 0.911 | 5.659 |
| SE | 0.005 | 0.006 | 0.004 | 0.004 | 0.004 | 0.008 | 0.010 | 0.007 | 0.008 | 0.535 | 0.298 | 0.405 | 0.204 | 0.010 | 0.018 |
| Min – Max Range | 1.267-1.380 | 1.098-1.254 | 0.986-1.070 | 0.231-0.304 | 0.803-0.873 | 1.915-2.112 | 0.507-0.704 | 0.253-0.394 | 1.464-1.691 | 104.546-119.481 | 20.000-26.230 | 73.478-84.451 | 62.319-65.964 | 0.817-1.014 | 5.435-5.814 |
| *zeteki* gyne  N=28  (10 nests) | **HW** | **HL** | **SL** | **EL** | **FL** | **ML** | **PL** | **PPL** | **GL** | **CI** | **EI** | **SI** | **FLI** | **WaL** | **TL** |
| Mean | 1.652 | 1.378 | 1.215 | 0.378 | 1.063 | 2.478 | 0.756 | 0.388 | 1.964 | 119.933 | 27.472 | 73.602 | 64.389 | 1.145 | 6.965 |
| SE | 0.011 | 0.010 | 0.007 | 0.003 | 0.008 | 0.012 | 0.016 | 0.005 | 0.015 | 0.423 | 0.220 | 0.255 | 0.240 | 0.017 | 0.043 |
| Min – Max Range | 1.462-1.800 | 1.258-1.520 | 1.110-1.310 | 0.333-0.399 | 0.925-1.121 | 2.272-2.584 | 0.539-0.873 | 0.338-0.424 | 1.656-2.090 | 115.980-126.677 | 25.507-29.203 | 71.111-75.923 | 62.050-67.238 | 0.886-1.267 | 6.072-7.296 |
| *mikro.* male  N=21  (5 nests) | **HW** | **HL** | **SL** | **EL** | **FL** | **ML** | **PL** | **PPL** | **GL** | **CI** | **EI** | **SI** | **FLI** | **WaL** | **TL** |
| Mean | 0.840 | 0.684 | 0.826 | 0.294 | 0.309 | 1.781 | 0.369 | 0.234 | 1.482 | 122.934 | 43.074 | 98.407 | 36.738 | 0.603 | 4.550 |
| SE | 0.004 | 0.005 | 0.004 | 0.003 | 0.003 | 0.012 | 0.006 | 0.004 | 0.031 | 0.956 | 0.403 | 0.618 | 0.369 | 0.008 | 0.035 |
| Min – Max Range | 0.817-0.893 | 0.620-0.732 | 0.788-0.873 | 0.282-0.310 | 0.279-0.338 | 1.671-1.859 | 0.338-0.437 | 0.197-0.266 | 1.239-1.746 | 115.437-134.084 | 40.057-45.858 | 91.713-103.427 | 33.259-40.000 | 0.535-0.684 | 4.196-4.759 |

**Table S4** *–* Partitioned morphometric measurements. Table of partitioned measurements for examined *Mycetomoellerius mikromelanos* sp. nov. (mikro.) and *M. zeteki* (zeteki) specimen by caste and species, including standard errors (SE), minimum and maximum standard measurements in millimeters, except for indices. The junior synonym of *M. zeteki* is included as “balboai” which are included in the entire *M. zeteki* partition.

| *zeteki* male  N=22  (6 nests) | HW | HL | SL | EL | FL | ML | PL | PPL | GL | CI | EI | SI | FLI | WaL | TL |
| --- | --- | --- | --- | --- | --- | --- | --- | --- | --- | --- | --- | --- | --- | --- | --- |
| Mean | 1.043 | 0.797 | 1.011 | 0.368 | 0.369 | 2.015 | 0.501 | 0.286 | 1.734 | 130.871 | 46.133 | 96.988 | 35.409 | 0.787 | 5.334 |
| SE | 0.009 | 0.007 | 0.011 | 0.003 | 0.003 | 0.020 | 0.010 | 0.006 | 0.035 | 1.012 | 0.346 | 0.822 | 0.427 | 0.012 | 0.059 |
| Min – Max Range | 0.957-1.140 | 0.746-0.874 | 0.929-1.100 | 0.338-0.399 | 0.338-0.411 | 1.830-2.168 | 0.422-0.591 | 0.253-0.342 | 1.603-2.242 | 125.826-142.322 | 42.893-48.225 | 86.822-102.687 | 31.579-39.443 | 0.704-0.929 | 4.984-5.902 |

**Table S4** *–* cont’d

| *mikro. m*ale  N=21  (5 nests) | HW | | HL | | SL | | EL | | FL | | ML | | PL | | PPL | | GL | | CI | | EI | | SI | | FLI | | WaL | | TL | |  |
| --- | --- | --- | --- | --- | --- | --- | --- | --- | --- | --- | --- | --- | --- | --- | --- | --- | --- | --- | --- | --- | --- | --- | --- | --- | --- | --- | --- | --- | --- | --- | --- |
| Mean | 0.840 | | 0.684 | | 0.826 | | 0.294 | | 0.309 | | 1.781 | | 0.369 | | 0.234 | | 1.482 | | 122.934 | | 43.074 | | 98.407 | | 36.738 | | 0.603 | | 4.550 | |  |
| SE | 0.004 | | 0.005 | | 0.004 | | 0.003 | | 0.003 | | 0.012 | | 0.006 | | 0.004 | | 0.031 | | 0.956 | | 0.403 | | 0.618 | | 0.369 | | 0.008 | | 0.035 | |  |
| Min – Max Range | 0.817-0.893 | | 0.620-0.732 | | 0.788-0.873 | | 0.282-0.310 | | 0.279-0.338 | | 1.671-1.859 | | 0.338-0.437 | | 0.197-0.266 | | 1.239-1.746 | | 115.437-134.084 | | 40.057-45.858 | | 91.713-103.427 | | 33.259-40.000 | | 0.535-0.684 | | 4.196-4.759 | |  |
| *zeteki* male  N=22  (6 nests) | | **HW** | | **HL** | | **SL** | | **EL** | | **FL** | | **ML** | | **PL** | | **PPL** | | **GL** | | **CI** | | **EI** | | **SI** | | **FLI** | | **WaL** | | **TL** | |
| Mean | | 1.043 | | 0.797 | | 1.011 | | 0.368 | | 0.369 | | 2.015 | | 0.501 | | 0.286 | | 1.734 | | 130.871 | | 46.133 | | 96.988 | | 35.409 | | 0.787 | | 5.334 | |
| SE | | 0.009 | | 0.007 | | 0.011 | | 0.003 | | 0.003 | | 0.020 | | 0.010 | | 0.006 | | 0.035 | | 1.012 | | 0.346 | | 0.822 | | 0.427 | | 0.012 | | 0.059 | |
| Min – Max Range | | 0.957-1.140 | | 0.746-0.874 | | 0.929-1.100 | | 0.338-0.399 | | 0.338-0.411 | | 1.830-2.168 | | 0.422-0.591 | | 0.253-0.342 | | 1.603-2.242 | | 125.826-142.322 | | 42.893-48.225 | | 86.822-102.687 | | 31.579-39.443 | | 0.704-0.929 | | 4.984-5.902 | |

**Table S4** *–* Partitioned morphometric measurements; continuted.
