# Supplementary figures and images for: Using an integrative taxonomic approach to delimit a sibling species, Mycetomoellerius mikromelanos sp. nov. (Formicidae: Attini: Attina)
Source: PeerJ. 2021 Jun 24;9:e11622. doi: 10.7717/peerj.11622 (PMC8236233; doi:10.7717/peerj.11622)

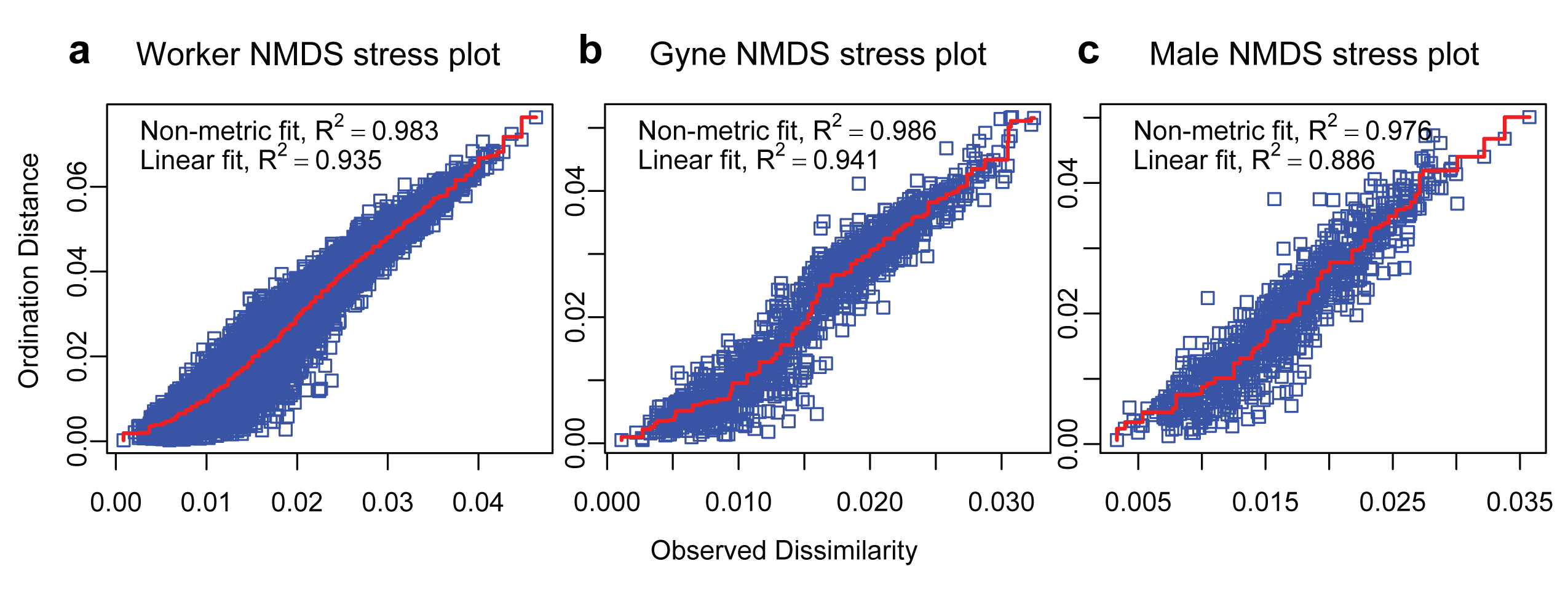

Supplement: Supplemental Information 6 [file peerj-09-11622-s006.png]

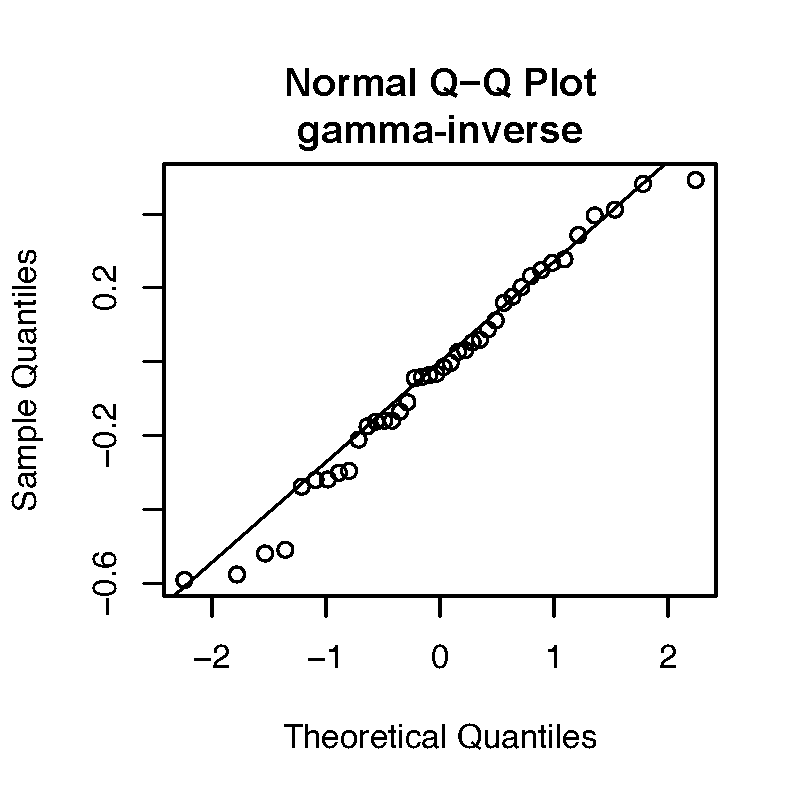

Supplement: Supplemental Information 7 [file peerj-09-11622-s007.png]

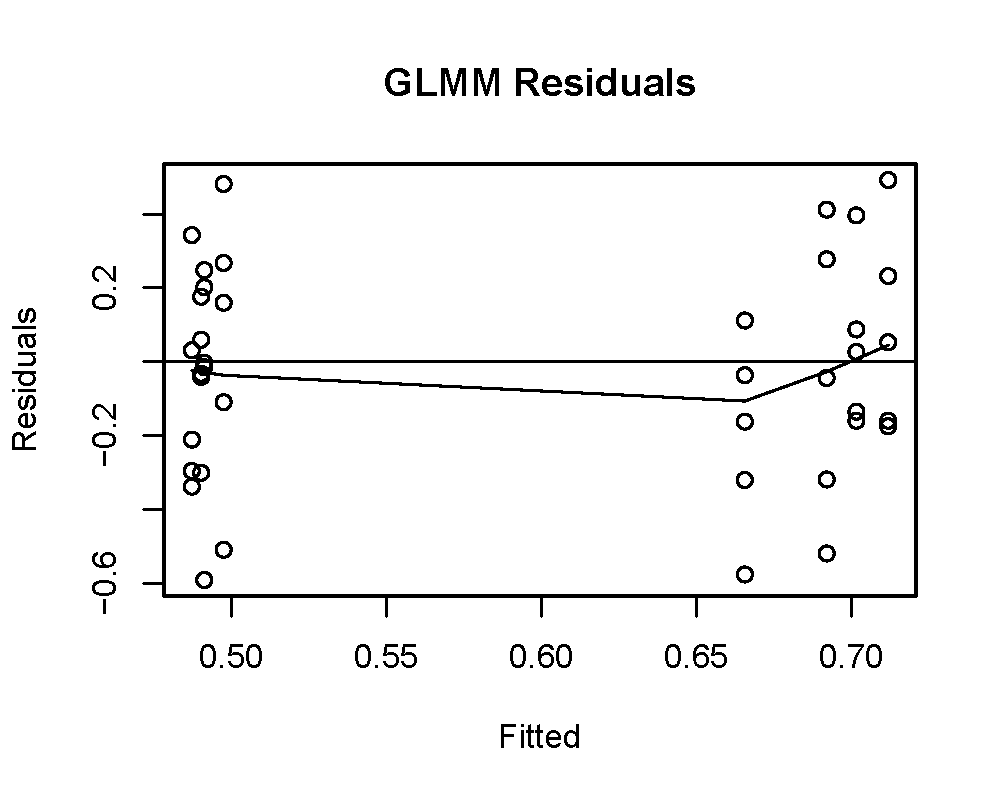

Supplement: Supplemental Information 8 [file peerj-09-11622-s008.png]

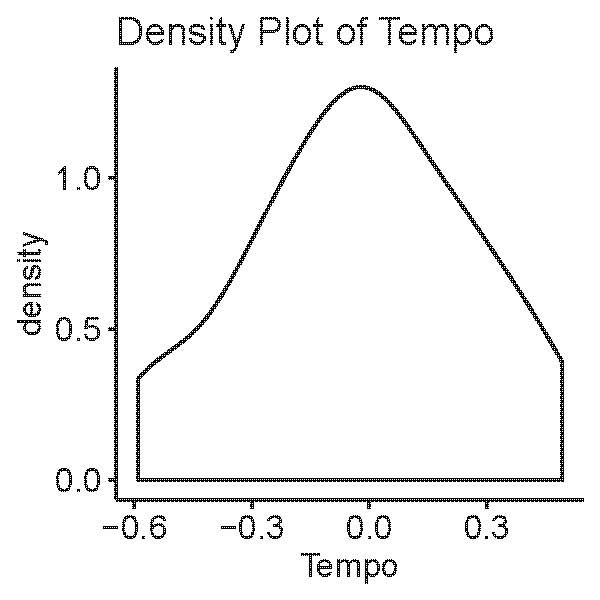

Supplement: Supplemental Information 9 [file peerj-09-11622-s009.png]

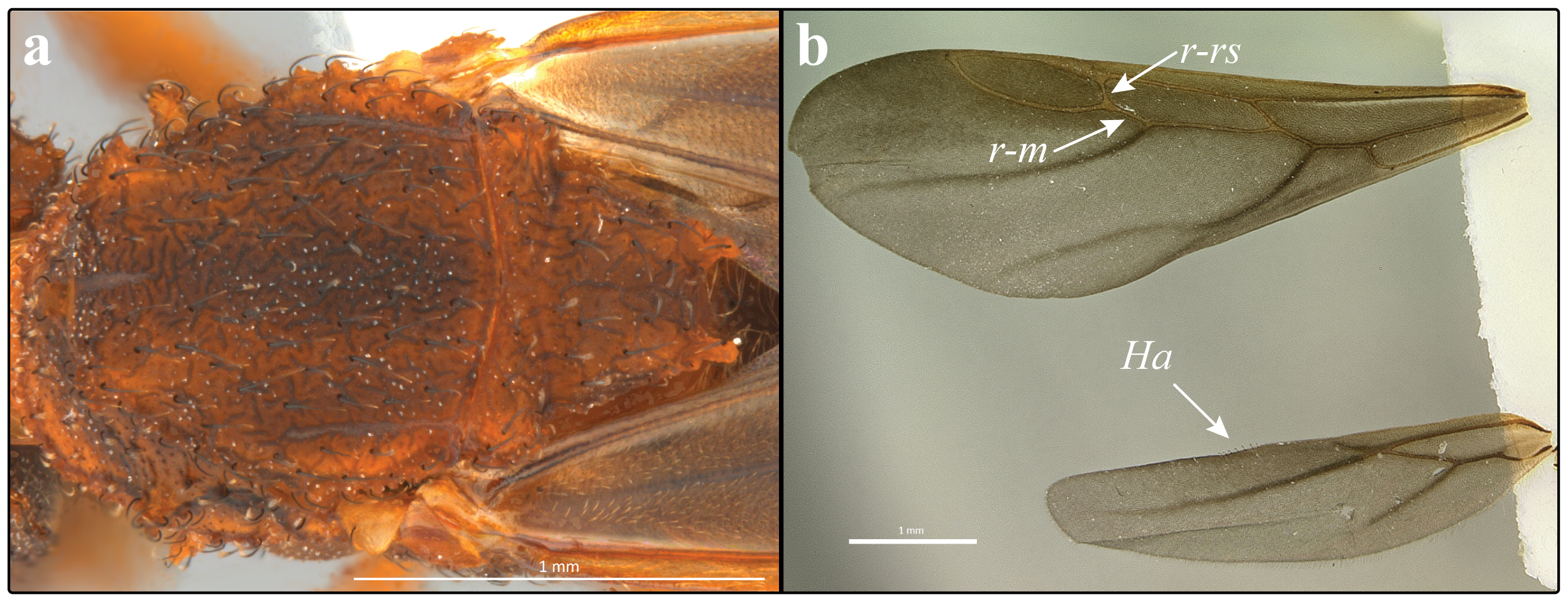

Supplement: Supplemental Information 10 — (a) Dorsal view of mesoscutum presenting random-reticulate sculpturing. (b) Wing veination: Ha = hamuli, r-rs = radius-radial sector; rs-m = radial sector-media. [file peerj-09-11622-s010.png]

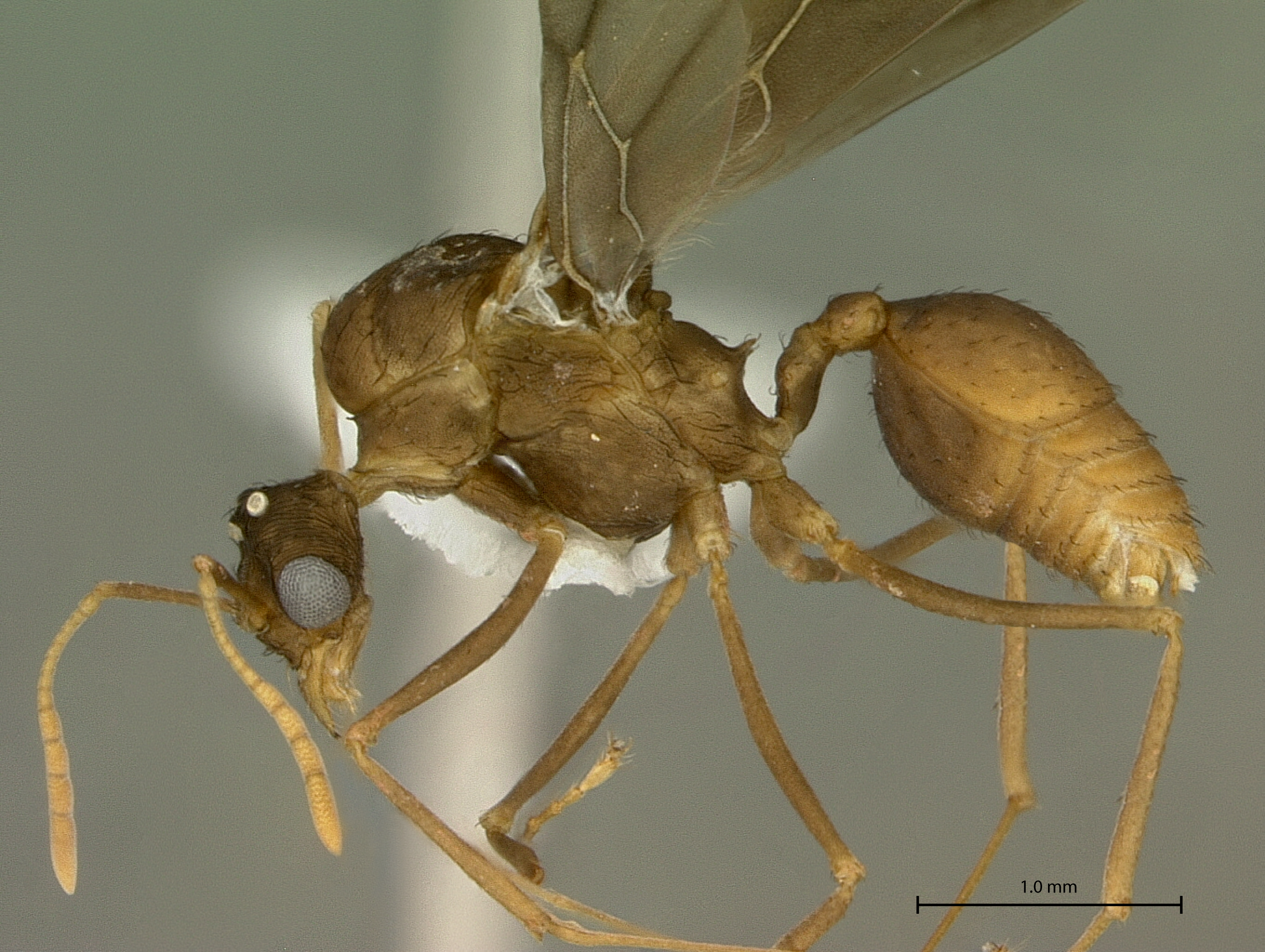

Supplement: Supplemental Information 11 — Lateral view providing an additional view of the the head capsule sculpturing. [file peerj-09-11622-s011.png]

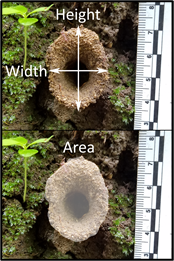

Supplement: Supplemental Information 12 [file peerj-09-11622-s012.png]

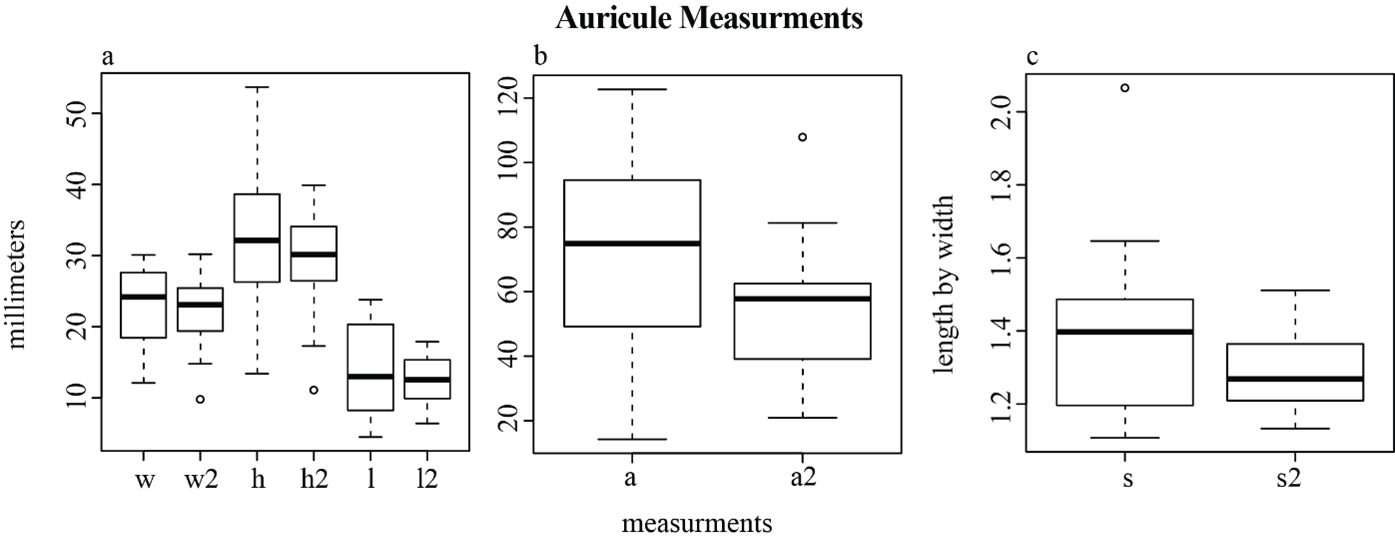

Supplement: Supplemental Information 13 — (A) Width = w, height = h, length = l, (B) area = a, (c) shape = s; measurements post knockdown are indicated with a 2 (e.g., w = pre-knockdown, w2 = post knockdown). [file peerj-09-11622-s013.png]

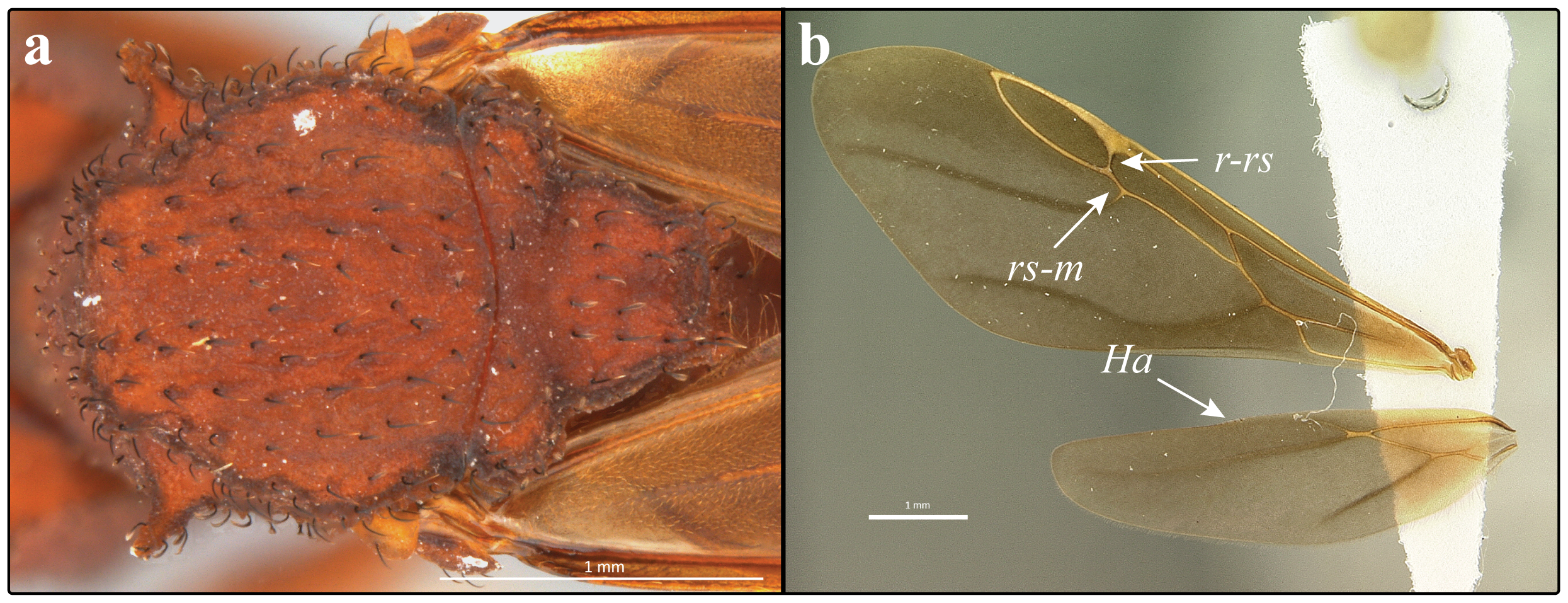

Supplement: Supplemental Information 14 — (A) Dorsal view of mesoscutum presenting random-reticulate sculpturing. (B) Wing veination: Ha = hamuli, r-rs = radius-radial sector; rs-m = radial sector-media. [file peerj-09-11622-s014.png]

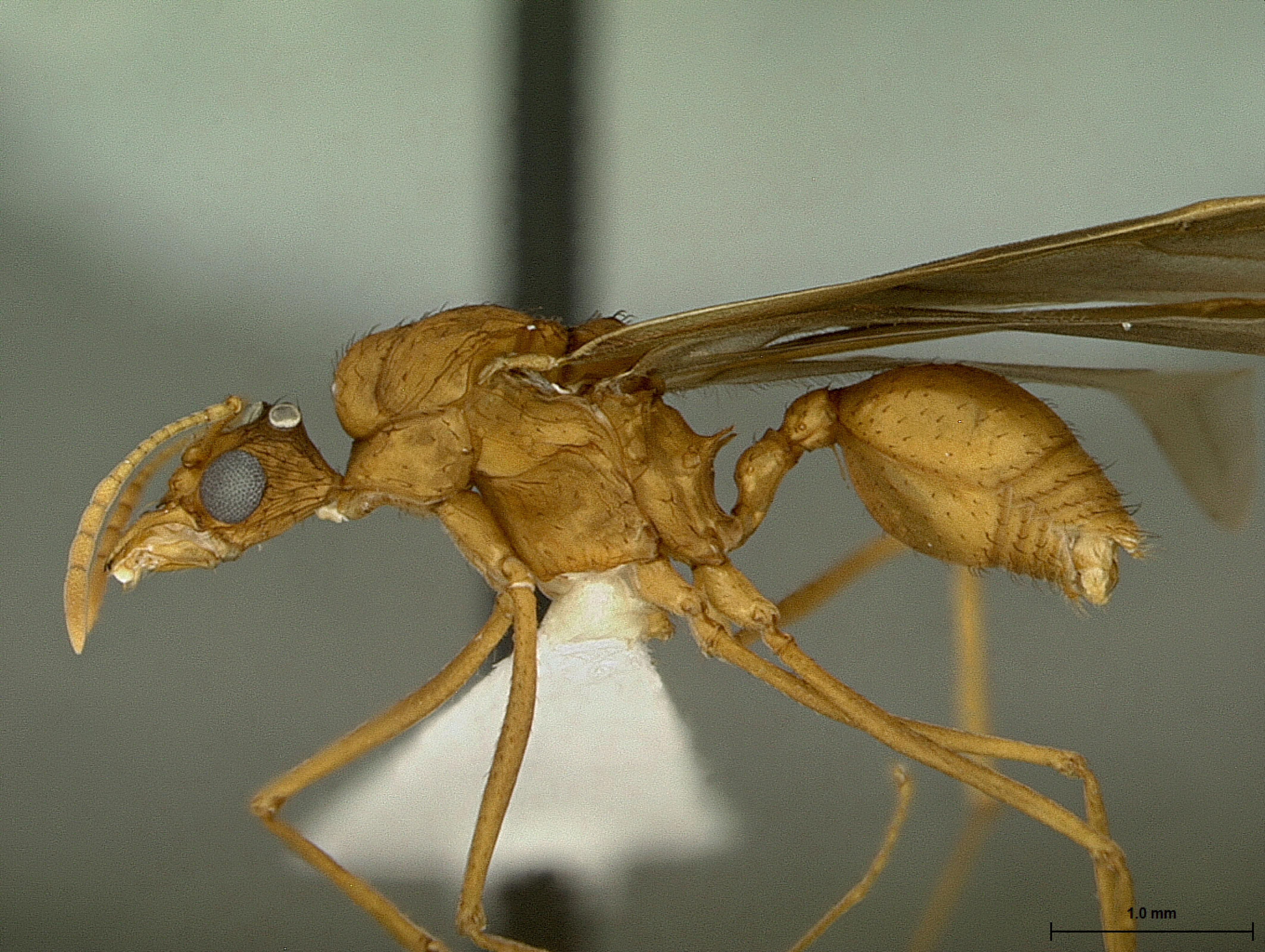

Supplement: Supplemental Information 15 — Lateral view providing an additional view of the the head capsule sculpturing. Notable as well is the variable ventral post-petiol process observed on some M. zeteki male specimens. [file peerj-09-11622-s015.png]

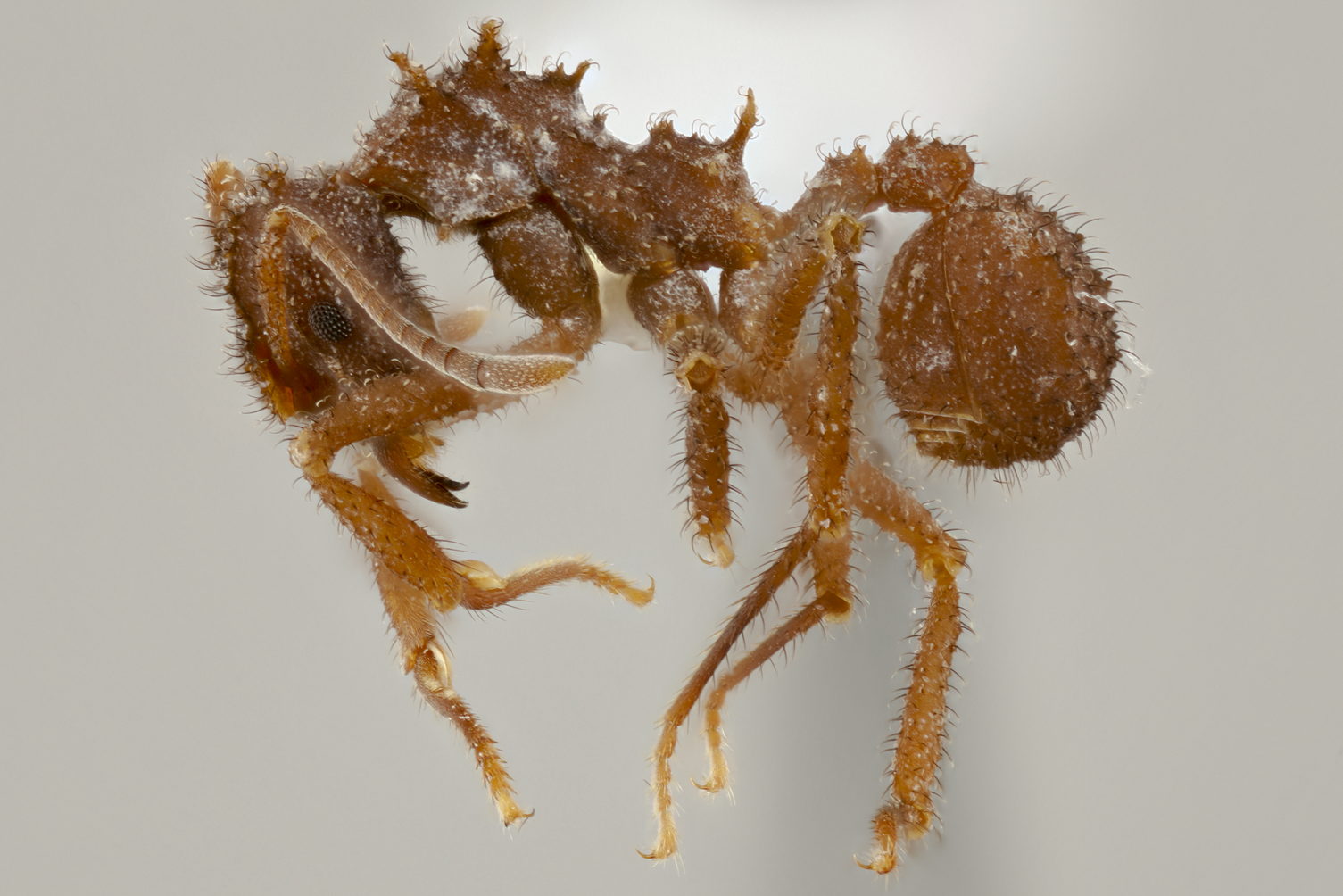

Supplement: Supplemental Information 16 [file peerj-09-11622-s016.png]

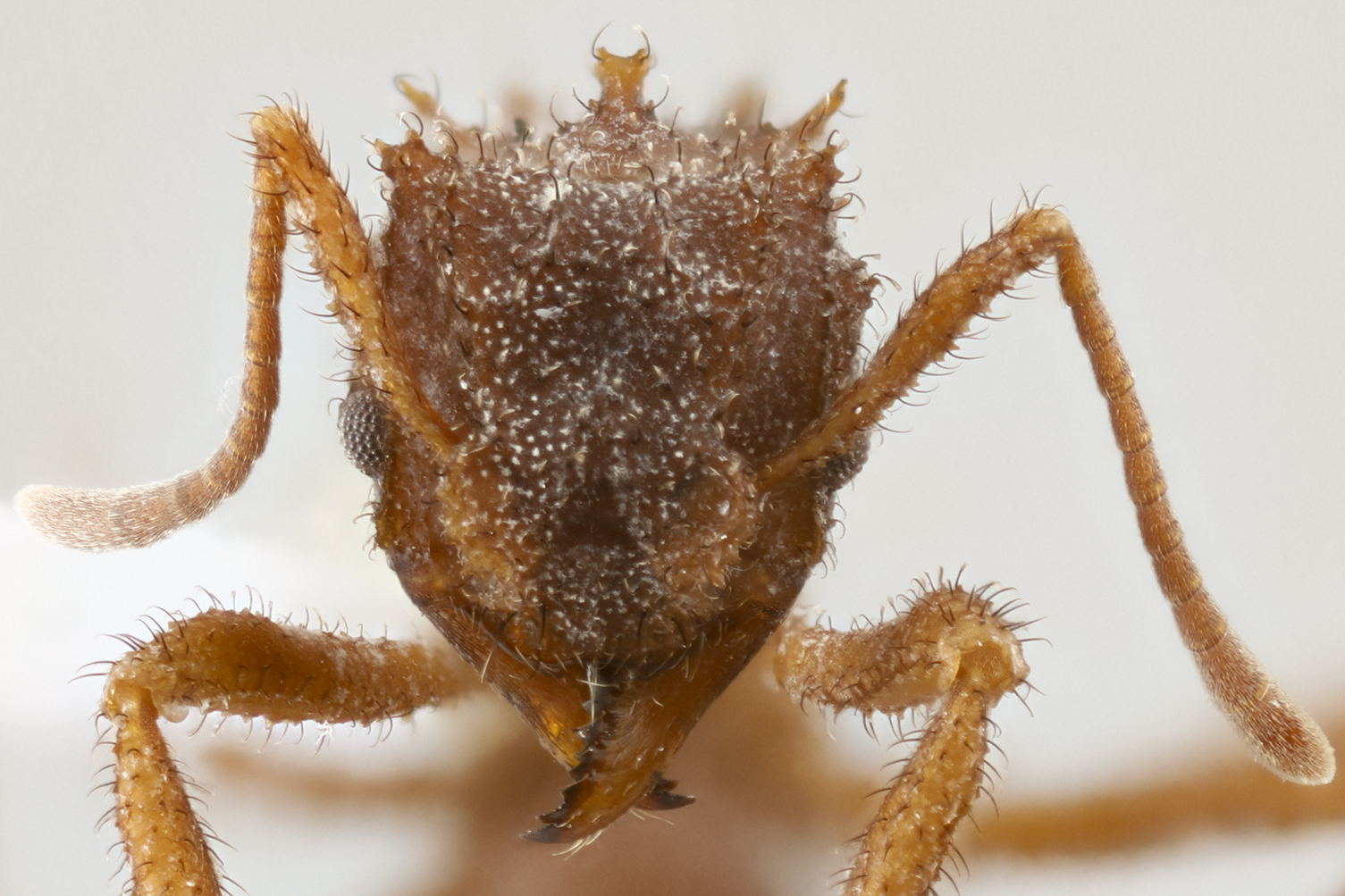

Supplement: Supplemental Information 17 [file peerj-09-11622-s017.png]
