# Supplementary material for: Using an integrative taxonomic approach to delimit a sibling species, Mycetomoellerius mikromelanos sp. nov. (Formicidae: Attini: Attina)
Source: PeerJ. 2021 Jun 24;9:e11622. doi: 10.7717/peerj.11622 (PMC8236233; doi:10.7717/peerj.11622)
Supplement: Supplemental Information 18 [file peerj-09-11622-s018.docx]

Supplemental Auricle data

We measured the height, width, length, area, of 16 auricles (Fig. S7). We also calculated shape by dividing width from height, which provides an approximate shape of the auricle. All measurements were calculated by photographing the nest entrance next to a forensics scale and measuring them digitally in ImageJ. After completing measurements, we removed the auricles of 16 nests from the embankment and returned seven days later to repeat the measurements.

Eleven of the sixteen colonies were used in a two-sided Welch’s two sample t-test, five colonies were removed due to difficulties gathering some measurements. Before performing our t-test we tested the normality and compared variances in the data using Shapiro-Wilk Normality test and F Test in R with the base stats package.

Results

All measurements except for shape taken before and after knockdown experiment met the expectation of normality and equal variance using a Shapiro-Wilk normality and F test. We then removed shape from further analyses. With our remaining measurements, the two-sided Welch two-sample T-test showed no statistically significant differences before and after auricle knock down experiments.

Nests were rebuilt to nearly the same width, height, area, and length (Fig. S7). However, shape variation was not normal before and after the knock down experiment. This may be due to an outlier in the first shape measurement (Fig.S1c). These results may be due to the natural variation, or the continued construction and maintenance, of the nest entrances. Regardless, auricles show some significant biological function not yet understood.
