# Supplementary material for: Using an integrative taxonomic approach to delimit a sibling species, Mycetomoellerius mikromelanos sp. nov. (Formicidae: Attini: Attina)
Source: PeerJ. 2021 Jun 24;9:e11622. doi: 10.7717/peerj.11622 (PMC8236233; doi:10.7717/peerj.11622)
Supplement: Supplemental Information 19 [file peerj-09-11622-s019.docx]

**Supplemental References**

Adams RMM, Jones TH, Jeter AW, De Fine Licht HH, Schultz TR, Nash DR (2012a) A comparative study of exocrine gland chemistry in *Trachymyrmex* and *Sericomyrmex* fungus-growing ants. Biochem Syst Ecol 40:91–97. https://doi.org/10.1016/j.bse.2011.10.011

Adams RMM, Liberti J, Illum AA, Jones TH, Nash DR, Boomsma JJ (2013) Chemically armed mercenary ants protect fungus-farming societies. Proc Natl Acad Sci 110:15752–15757. https://doi.org/10.1073/pnas.1311654110

Adams RMM, Shah K, Antonov LD, Mueller UG (2012b) Fitness consequences of nest infiltration by the mutualist-exploiter *Megalomyrmex adamsae*. Ecol Entomol 37:453–462. https://doi.org/10.1111/j.1365-2311.2012.01384.x

Andersen SB, Hansen LH, Sapountzis P, Sørensen SJ, Boomsma JJ (2013) Specificity and stability of the *Acromyrmex*-*Pseudonocardia* symbiosis. Mol Ecol 22:4307–4321. https://doi.org/10.1111/mec.12380

Armitage SAO, Wcislo WT, Boomsma JJ (2012) An evaluation of the possible adaptive function of fungal brood covering by Attine ants. Evolution (N Y) 66:1966–1975. https://doi.org/10.5061/dryad.r36d6k6t

Baer B, Boomsma JJ (2004) Male reproductive investment and queen mating-frequency in fungus-growing ants. Behav Ecol 15:426–432. https://doi.org/10.1093/beheco/arh025

Baer B, Dijkstra MB, Mueller UG, Nash DR, Boomsma JJ (2009) Sperm length evolution in the fungus-growing ants. Behav Ecol 20:38–45. https://doi.org/10.1093/beheco/arn112

Birnbaum SSL, Gerardo NM (2016) Patterns of specificity of the pathogen *Escovopsis* across the fungus-growing ant symbiosis. Am Nat 188:52–65. https://doi.org/10.1086/686911

Bolton B (1995) A new general catalogue of the ants of the world. Harvard University Press, Cambridge, Massachusetts

Boya CA, Fernández-Marín H, Mejiá LC, Spadafora C, Dorrestein PC, Gutiérrez M (2017) Imaging mass spectrometry and MS/MS molecular networking reveals chemical interactions among cuticular bacteria and pathogenic fungi associated with fungus-growing ants. Sci Rep 7:1–13. https://doi.org/10.1038/s41598-017-05515-6

Brandão CRF, Mayhé-Nunes AJ (2007) A phylogenetic hypothesis for the *Trachymyrmex* species groups, and the transition from fungus-growing to leaf-cutting in the Attini. Mem Am Entomol Inst 80:73–87. https://doi.org/10.1533/9781845696382.2.267

Brandão CRF, Mayhé-Nunes AJ (2008) A new species of the fungus-farming ant genus *Mycetagroicus* Brandão & Mayhé-Nunes (Hymenoptera, Formicidae, Attini). Rev Bras Entomol 52:349–352. https://doi.org/10.1590/S0085-56262008000300006

Brandão CRF, Mayhé-Nunes AJ (2001) A new fungus-growing ant genus, *Mycetagroicus* gen. n., with the description of three new species and comments on the monophyly of the Attini (Hymenoptera: Formicidae). Sociobiology 38:639–665

Cafaro MJ, Currie CR (2005) Phylogenetic analysis of mutualistic filamentous bacteria associated with fungus-growing ants. Can J Microbiol 51:441–446. https://doi.org/10.1139/w05-023

Cafaro MJ, Poulsen M, Little AEF, Price SL, Gerardo NM, Wong B, Stuart AE, Larget B, Abbot P, Currie CR (2011) Specificity in the symbiotic association between fungus-growing ants and protective *Pseudonocardia* bacteria. Proc R Soc B Biol Sci 278:1814–1822. https://doi.org/10.1098/rspb.2010.2118

Currie CR, Mueller UG, Malloch D (1999) The agricultural pathology of ant fungus gardens. Proc Natl Acad Sci U S A 96:7998–8002

Currie CR, Poulsen M, Mendenhall J, Boomsma JJ, Billen J (2006) Coevolved crypts and exocrine glands support mutualistic bacteria in fungus-growing ants. Science (80- ) 311:81–83. https://doi.org/10.1126/science.1119744

Currie CR, Wong B, Stuart AE, Schultz TR, Rehner SA, Mueller UG, Sung GH, Spatafora JW, Straus NA (2003) Ancient tripartite coevolution in the attine ant-microbe symbiosis. Science (80- ) 299:386–388. https://doi.org/10.1126/science.1078155

de Andrade ML (2003) First descriptions of two new amber species of *Cyphomyrmex* from Mexico and the Dominican Republic. Beiträge zur Entomol 53:131–139. https://doi.org/10.21248/contrib.entomol.53.1.131-139

De Fine Licht HH, Boomsma JJ (2014) Variable interaction specificity and symbiont performance in Panamanian *Trachymyrmex* and *Sericomyrmex* fungus-growing ants. BMC Evol Biol 14:244. https://doi.org/10.1186/s12862-014-0244-6

De Fine Licht HH, Boomsma JJ, Tunlid A (2014) Symbiotic adaptations in the fungal cultivar of leaf-cutting ants. Nat Commun 5:1–10. https://doi.org/10.1038/ncomms6675

De Souza DJ, Soares IMF, Della Lucia TMC (2007) *Acromyrmex ameliae* sp. n. (Hymenoptera: Formicidae): a new social parasite of leaf-cutting ants in Brazil. Insect Sci 14:251–257. https://doi.org/10.1111/j.1744-7917.2007.00151.x

Den Boer SPA, Baer B, Boomsma JJ, Smith JM, Biol T (2010) Seminal fluid mediates ejaculate competition in social insects. Science (80- ) 327:1506–1509. https://doi.org/10.1126/science.1184709

Dijkstra MB, Boomsma JJ (2008) Sex allocation in fungus-growing ants: Worker or queen control without symbiont-induced female bias. Oikos 117:1892–1906. https://doi.org/10.1111/j.1600-0706.2008.16822.x

Donoso DA (2014) Assembly mechanisms shaping tropical litter ant communities. Ecography (Cop) 37:490–499. https://doi.org/10.1111/j.1600-0587.2013.00253.x

Elizondo Wallace DE, Vargas Asensio JG, Pinto Tomás AA (2014) Correlation between virulence and genetic structure of *Escovopsis* strains from leaf-cutting ant colonies in Costa Rica. Microbiology 160:1727–1736. https://doi.org/10.1099/mic.0.073593-0

Fernández-Marín H, Bruner G, Gomez EB, Nash DR, Boomsma JJ, Wcislo WT (2013) Dynamic disease management in *Trachymyrmex* fungus-growing ants (Attini: Formicidae). Am Nat 181:571–582. https://doi.org/10.1086/669664

Fernández-Marín H, Nash DR, Higginbotham S, Estrada C, Van Zweden JS, D’Ettorre P, Wcislo WT, Boomsma JJ (2015) Functional role of phenylacetic acid from metapleural gland secretions in controlling fungal pathogens in evolutionarily derived leaf-cutting ants. Proc R Soc B Biol Sci 282:1–9. https://doi.org/10.1098/rspb.2015.0212

Fernández-Marín H, Zimmerman JK, Nash DR, Boomsma JJ, Wcislo WT (2009) Reduced biological control and enhanced chemical pest management in the evolution of fungus farming in ants. Proc R Soc B Biol 276:2263–2269. https://doi.org/10.1098/rspb.2009.0184

Fernández-Marín H, Zimmerman JK, Rehner SA, Wcislo WT (2006) Active use of the metapleural glands by ants in controlling fungal infection. Proc R Soc B Biol 273:1689–1695

Fernández-Marín H, Zimmerman JK, Wcislo WT (2004) Ecological traits and evolutionary sequence of nest establishment in fungus-growing ants (Hymenoptera, Formicidae, Attini). Biol J Linn Soc 81:39–48. https://doi.org/10.1111/j.1095-8312.2004.00268.x

Fjerdingstad EJ, Crozier RH (2006) The evolution of worker caste diversity in social insects. Am Nat 167:390–400. https://doi.org/10.1086/499545

Fontenla JL (1995) Nueva especie de *Atta* (Hymenoptera: Formicidae) del archipiélago cubano. Avicennia 3:77–86

Frost CL, Fernández-Marín H, Smith JE, Hughes WOH (2010) Multiple gains and losses of *Wolbachia* symbionts across a tribe of fungus-growing ants. Mol Ecol 19:4077–4085. https://doi.org/10.1111/j.1365-294X.2010.04764.x

Hughes WOH, Pagliarini R, Madsen HB, Dijkstra MB, Boomsma JJ (2008) Antimicrobial defense shows an abrupt evolutionary transition in the fungus-growing ants. Evolution 62:1252–7. https://doi.org/10.1111/j.1558-5646.2008.00347.x

Ješovnik A, González VL, Schultz TR (2016) Phylogenomics and divergence dating of fungus-farming ants (Hymenoptera: Formicidae) of the genera *Sericomyrmex* and *Apterostigma*. PLoS One 11:1–18. https://doi.org/10.1371/journal.pone.0151059

Ješovnik A, Schultz TR (2017) Revision of the fungus-farming ant genus *Sericomyrmex* Mayr (Hymenoptera, Formicidae, Myrmicinae). Zookeys 670:1–109. https://doi.org/10.3897/zookeys.670.11839

Ješovnik A, Sosa-Calvo J, Lloyd MW, Branstetter MG, Fernández F, Schultz TR (2017) Phylogenomic species delimitation and host-symbiont coevolution in the fungus-farming ant genus *Sericomyrmex* Mayr (Hymenoptera: Formicidae): ultraconserved elements (UCEs) resolve a recent radiation. Syst Entomol 42:523–542. https://doi.org/10.1111/syen.12228

Kaspari M, Donoso D, Lucas JA, Zumbusch T, Kay AD (2012) Using nutritional ecology to predict community structure: a field test in Neotropical ants. Ecosphere 3:1–15

Klingenberg C, Brandão CRF (2009) Revision of the fungus-growing ant genera *Mycetophylax* Emery and *Paramycetophylax* Kusnezov rev. stat., and description of *Kalathomyrmex* n. gen. (Formicidae: Myrmicinae: Attini). Zootaxa 2052:1–31

Kooij PW, Aanen DK, Schiøtt M, Boomsma JJ (2015) Evolutionarily advanced ant farmers rear polyploid fungal crops. J Evol Biol 28:1911–1924. https://doi.org/10.1111/jeb.12718

Lattke JE (1997) Revisión del género *Apterostigma* Mayr (Hymenoptera: Formicidae). Arq Zool Sao Paulo 34:121–221

Lattke JE (1999) A new species of fungus-growing ant and its implications for attine phylogeny (Hymenoptera: Formicidae). Syst Entomol 24:1–6

Liberti J, Sapountzis P, Hansen LH, Sørensen SJ, Adams RMM, Boomsma JJ (2015) Bacterial symbiont sharing in Megalomyrmex social parasites and their fungus-growing ant hosts. Mol Ecol 24:3151–3169. https://doi.org/10.1111/mec.13216

Little AE, Currie CR (2009) Parasites may help stabilize cooperative relationships. BMC Evol Biol 9:1–9. https://doi.org/10.1186/1471-2148-9-124

Little AEF, Murakami T, Mueller UG, Currie CR (2003) The infrabuccal pellet piles of fungus-growing ants. Naturwissenschaften 90:558–562. https://doi.org/10.1007/s00114-003-0480-x

Little AEF, Murakami T, Mueller UG, Currie CR (2006) Defending against parasites: fungus-growing ants combine specialized behaviours and microbial symbionts to protect their fungus gardens. Biol Lett 2:12–16. https://doi.org/doi:10.1098/rsbl.2005.0371

Longino JT, Colwell RK (2011) Density compensation, species composition, and richness of ants on a neotropical elevational gradient. Ecosphere 2:1–20. https://doi.org/10.1890/ES10-00200.1

Mackay WP (1998) Dos especies nuevas de hormigas de la tribu Attini de Costa Rica y México: *Mycetosoritis vinsoni* y *Mycocepurus curvispinosus* (Hymenoptera: Formicidae). Rev Biol Trop 46:421–426. https://doi.org/10.1063/1.1733542

Mackay WP, Mackay EE (1997) Una nueva especie de hormiga del género Trachymyrmex (Hymenoptera: Formicidae) del Estado de Chihuahua, México. Sociobiology 30:43–49

Mackay WP, Serna F (2010) Two new species of the strigatus species complex of the ant genus *Cyphomyrmex* (Hymenoptera: Formicidae) from Costa Rica and Panamá. J Hymenopt Res 19:44–50

Mangone DM, Currie CR (2007) Garden substrate preparation behaviours in fungus-growing ants. Can Entomol 139:841–849. https://doi.org/http://esc-sec.org/canent1.htm

Mayhé-Nunes AJ (1995) Sinopse do gênero *Mycetarotes* (Hym., Formicidae), com a descrição de duas especies novas. Boletín Entomol Venez 10:197–205

Mayhé-Nunes AJ, Brandão CRF (2007) Revisionary studies on the attine ant genus *Trachymyrmex* Forel. Part 3: the Jamaicensis group (Hymenoptera: Formicidae). Zootaxa 1444:1–21

Mayhé-Nunes AJ, Brandão CRF (2002) Revisionary studies on the Attine ant genus Trachymyrmex Forel. Part 1: definition of the genus and the *Opulentus* group (Hymenoptera: Formicidae). Sociobiology 40:667–698

Mayhé-Nunes AJ, Brandão CRF (2005) Revisionary studies on the Attine ant genus *Trachymyrmex* Forel. Part 2: the *Iheringi* group (Hymenoptera: Formicidae). Sociobiology 45:271–305

Mueller UG, Dash D, Rabeling C, Rodrigues A (2008) Coevolution between attine ants and actinomycete bacteria: a reevaluation. Evolution (N Y) 62:2894–2912. https://doi.org/10.1111/j.1558-5646.2008.00501.x

Nygaard S, Hu H, Li C, Schiøtt M, Chen Z, Yang Z, Xie Q, Ma C, Deng Y, Dikow R, Rabeling C, Nash DR, Wcislo WT, Brady SG, Schultz TR, Zhang G, Boomsma JJ (2016) Reciprocal genomic evolution in the ant-fungus agricultural symbiosis. Nat Commun 7:1–9. https://doi.org/10.1038/ncomms12233

Pérez-Ortega B, Fernández-Marín H, Loiácono MS, Galgani P, Wcislo WT (2010) Biological notes on a fungus-growing ant, *Trachymyrmex* cf. *zeteki* (Hymenoptera, Formicidae, Attini) attacked by a diverse community of parasitoid wasps (Hymenoptera, Diapriidae). Insectes Soc 57:317–322. https://doi.org/10.1007/s00040-010-0086-1

Poulsen M, Currie CR (2010) Symbiont interactions in a tripartite mutualism: exploring the presence and impact of antagonism between two fungus-growing ant mutualists. PLoS One 5:e8748. https://doi.org/10.1371/journal.pone.0008748

Poulsen M, Erhardt DP, Molinaro DJ, Lin T-L, Currie CR (2007) Antagonistic bacterial interactions help shape host-symbiont dynamics within the fungus-growing ant-microbe mutualism. PLoS One 2:1–15. https://doi.org/10.1371/journal.pone.0000960

Rabeling C, Bacci M (2010) A new workerless inquiline in the lower Attini (Hymenoptera: Formicidae), with a discussion of social parasitism in fungus-growing ants. Syst Entomol 35:379–392. https://doi.org/10.1111/j.1365-3113.2010.00533.x

Rabeling C, Cover SP, Johnson RA, Mueller UG (2007) A review of the North American species of the fungus-gardening ant genus Trachymyrmex (Hymenoptera: Formicidae). Zootaxa 1–53. https://doi.org/http://www.mapress.com/zootaxa/

Rabeling C, Schultz TR, Bacci M, Bollazzi M (2015) *Acromyrmex charruanus*: a new inquiline social parasite species of leaf-cutting ants. Insectes Soc 62:335–349. https://doi.org/10.1007/s00040-015-0406-6

Sánchez-Peña SR, Chacón-Cardosa MC, Canales-del-Castillo R, Ward L, Resendez-Pérez D (2017) A new species of *Trachymyrmex* (Hymenoptera, Formicidae) fungus-growing ant from the Sierra Madre Oriental of northeastern Mexico. Zookeys 2017:73–94. https://doi.org/10.3897/zookeys.706.12539

Sapountzis P, Zhukova M, Shik JZ, Schiott M, Boomsma JJ (2018) Reconstructing the functions of endosymbiotic mollicutes in fungus-growing ants. Elife 7:1–31. https://doi.org/10.7554/eLife.39209

Schultz TR (2007) The fungus-growing ant genus *Apterostigma* in Dominican amber. Mem Am Entomol Inst 80:425–436

Schultz TR, Bekkevold D, Boomsma JJ (1998) *Acromyrmex insinuator* new species: an incipient social parasite of fungus-growing ants. Insectes Soc 45:457–471. https://doi.org/10.1007/s000400050101

Schultz TR, Brady SG (2008) Major evolutionary transitions in ant agriculture. Proc Natl Acad Sci 105:5435–5440. https://doi.org/10.1073/pnas.0711024105

Schultz TR, Solomon SA, Mueller UG, Villesen P, Boomsma JJ, Adams RMM, Norden B (2002) Cryptic speciation in the fungus-growing ants *Cyphomyrmex longiscapus* Weber and *Cyphomyrmex muelleri* Schultz and Solomon, new species (Formicidae, Attini). Insectes Soc 49:331–343. https://doi.org/10.1007/PL00012657

Scott JJ, Weskin MK, Cooper M, Mueller UG (2009) Polymorphic microsatellite markers for the symbiotic fungi cultivated by leaf cutter ants (Attini, Formicidae). Mol Ecol Resour 9:1391–1394. https://doi.org/10.3182/20120912-3-BG-2031.00041

Seal JN (2009) Scaling of body weight and fat content in fungus-gardening ant queens: does this explain why leaf-cutting ants found claustrally? Insectes Soc 56:135–141. https://doi.org/10.1007/s00040-009-0002-8

Seid MA, Castillo A, Wcislo WT (2011) The allometry of brain miniaturization in ants. Brain Behav Evol 77:5–13. https://doi.org/10.1159/000322530

Semenova TA, Hughes DP, Boomsma JJ, Schiøtt M (2011) Evolutionary patterns of proteinase activity in attine ant fungus gardens. BMC Microbiol 11:1–11. https://doi.org/10.1186/1471-2180-11-15

Sen R, Ishak HD, Estrada D, Dowd SE, Hong E, Mueller UG (2009) Generalized antifungal activity and 454-screening of Pseudonocardia and Amycolatopsis bacteria in nests of fungus-growing ants. Proc Natl Acad Sci 106:17805–17810. https://doi.org/10.1073/pnas.0904827106

Shik JZ, Santos JC, Seal JN, Kay A, Mueller UG, Kaspari M (2014) Metabolism and the rise of fungus cultivation by ants. Am Nat 184:364–373. https://doi.org/10.1086/677296

Silva RR, Feitosa RSM, Eberhardt F (2007) Reduced ant diversity along a habitat regeneration gradient in the southern Brazilian Atlantic Forest. For Ecol Manage 240:61–69. https://doi.org/10.1016/j.foreco.2006.12.002

Solomon SE, Rabeling C, Sosa-Calvo J, Lopes CT, Rodrigues A, Vasconcelos HL, Bacci M, Mueller UG, Schultz TR (2019) The molecular phylogenetics of *Trachymyrmex* Forel ants and their fungal cultivars provide insights into the origin and coevolutionary history of ‘higher-attine’ ant agriculture. Syst Entomol 44:939–956. https://doi.org/10.1111/syen.12370

Sosa-Calvo J, Schultz TR (2010) Three remarkable new fungus-growing ant species of the genus *Myrmicocrypta* (Hymenoptera: Formicidae), with a reassessment of the characters that define the genus and its position within the attini. Ann Entomol Soc Am 103:181–195. https://doi.org/10.1603/AN09108

Sosa-Calvo J, Schultz TR, Brandão CRF, Klingenberg C, Feitosa RM, Rabeling C, Bacci MJ, Lopes CT, Vasconcelos HL (2013) *Cyatta abscondita*: taxonomy, evolution, and natural history of a new fungus-farming ant genus from Brazil. PLoS One 8:1–20. https://doi.org/10.1371/journal.pone.0080498

Sosa-Calvo J, Schultz TR, JeŠovnik A, Dahan RA, Rabeling C (2018) Evolution, systematics, and natural history of a new genus of cryptobiotic fungus-growing ants. Syst Entomol 43:549–567. https://doi.org/10.1111/syen.12289

Taerum SJ, Cafaro MJ, Little AEF, Schultz TR, Currie CR (2007) Low host-pathogen specificity in the leaf-cutting ant-microbe symbiosis. Proc R Soc B Biol Sci 274:1971–1978. https://doi.org/10.1098/rspb.2007.0431

Valdés-Rodríguez S, Chacón de Ulloa P, Armbrecht I (2014) Especies de hormigas del suelo en el Parque Nacional Natural Gorgona, Pacífico Colombiano. Rev Biol Trop 62:265. https://doi.org/10.15517/rbt.v62i0.16340

Vergara-Navarro E, Serna F (2013) A checklist of the ants (Hymenoptera: Formicidae) of the department of Antioquia, Colombia and new records for the country. Agron Colomb 31:324–342

Villesen P, Boomsma JJ (2003) Patterns of male parentage in the fungus-growing ants. Behav Ecol Sociobiol 53:246–253. https://doi.org/10.1007/s00265-002-0577-9

Villesen P, Gertsch PJ, Boomsma JJ (2002a) Microsatellite primers for fungus growing ants. Mol Ecol Notes 2:320–322. https://doi.org/10.1046/j.l471-8278

Villesen P, Murakami T, Schultz TR, Boomsma JJ (2002b) Identifying the transition between single and multiple mating of queens in fungus-growing ants. Proc R Soc London B Biol 269:1541–8. https://doi.org/10.1098/rspb.2002.2044

Weber NA (1940) The biology of the fungus-growing ants. Part VI. Key to *Cyphomyrmex*, new Attini and a new guest ant. Rev Entomol Rio Janeiro 11:406–427. https://doi.org/10.5281/zenodo.25008

Wetterer JK, Schultz TR, Meier R (1998) Phylogeny of fungus-growing ants (Tribe Attini) based on mtDNA sequence and morphology. Mol Phylogenet Evol 9:42–47. https://doi.org/10.1006/mpev.1997.0466

Zhang MM, Poulsen M, Currie CR (2007) Symbiont recognition of mutualistic bacteria by *Acromyrmex* leaf-cutting ants. ISME J 1:313–320. https://doi.org/10.1038/ismej.2007.41
